# CFD simulation on optimum material to fabricate the counter flow of Ranque–Hilsch vortex tube

**DOI:** 10.1038/s41598-022-19779-0

**Published:** 2022-09-28

**Authors:** K. Kiran Kumar Rao, A. Ramesh, Chennabasappa Hampali

**Affiliations:** 1 School of Mechanical Engineering, Reva University, Bangalore, 560064 India; 2 BIT Institute of Technology, Hindupur, A.P 515201 India; 3 School of Mechanical Engineering, Reva University, Bangalore, India

**Keywords:** Energy science and technology, Engineering

## Abstract

The basic objective of the current investigation by using CFD simulation is to select the appropriate material to fabricate Vortex tubes in meteorological conditions, which can improve efficiency and performance. The simulation results of vortex tubes were used in obtaining the performance for various materials such as copper, stainlessteel, brass, PVC, CPVC, acrylic, nylon, and bronze. The ratio of length of the hot tube and inner diameter was chosen as (L/D) 40 with the consistent inner diameter of the hot tube as 15 mm. Compressed air is passed through an inlet pipe between 2 and 12 bars in the step of 2 bars. 2D CFD was developed and analyzed with Ansys Fluent to access the performance of the vortex tube. Finally, after evaluating all simulation values for eight materials for performance depending upon their physical properties varies in the sequence from copper–bronze–brass–stainless steel–CPVC–PVC–acrylic–nylon material.

## Introduction

In recent times the major concern of any research or development is to protect the environment Design and development of new systems that promote ecological balance, conservation of energy and other resources, and recycling of waste are key areas in the current scenario. Today, environmental safety has become a major issue for both industry and mankind. This article aims to achieve better performance; increased efficiency of such an environmentally friendly system called vortex tube used for industrial spot cooling and process cooling equipment such as spot cooling, welding cooling, plastic cutting, extrusion cooling, food cooling, etc. Refrigeration systems use gases and liquids which cause the reduction of the ozone layer or promote global warming. Efforts have been made to create environmentally friendly refrigeration systems.

### Literature review

The vortex tube was invented in 1933 by George Ranque and then rebuilt in 1947 by Rudolf Hilsch^1^. To commemorate their development, this tube is popularly acknowledged as the Ranque–Hilsch vortex pipe (RHVT). There are different studies made on the effects of temperature (energy), leaving the vortex tube. Hilsch^2^ first studied the path of the vortex tube and the constitutional friction that led to the expansion of the vortex tube. The latest development uses CFD analysis to clarify the basic principles backing the energy dissolution process of the vortex tube. Bruun et al.^3^ worked on the velocity of the gas in a counter flow vortex tube and resolve the axial variation of the flow abundance; assessments were also undertaken on the cross-section of VT. Soni et al.^4^ reported that the performance of the vortex tube, the standard k–ϵ type performs better than the RNG k–ϵ type by using the CFD simulation fluent software. Ahlborn et al.^5,6^ observed that the return flow at the mean point of the vortex tube was higher than the cold mass flow appearing at the cold end side. Also, they noticed that the secondary circulation zone was introduced into the main vortex flow, which moves fluid from the hot end in the reverse direction and forms a core inside the outer vortex flow region. The energy separation described by. Abdelghany et al.^7^ explained the performance of the tube which is deeply influenced by the vortex tube L/D ratio and when it operated at various cold mass fractions. The maximum COP developed at cold mass fraction was found to be 0.64. Baghdad et al.^8^ used CFD in energy analysis to define the energy separation process and flow of fluid, heat transfer was compared using CO_2_ and air as working fluids. Pouraria et al.^9^ worked on a numerical simulation carried out using Fluent to solve the two-dimensional axis-symmetric swirl model of the vortex tube.

Liang et al.^10^ investigated by using CFD simulation the geometric structure of hot tubes; changing velocity and temperature on convergent and divergent hot tubes by 3°–5°. Increased angles at the hot tubes caused to decrease in the throat temperature and an increase in velocity. The maximum heat transfer is reached by a hot tube with the angle set at 40°. Khait et al.^11^ proposed flow formation with a transonic nozzle designed; explained the energy separation and energy efficiency process by supersonic speed; the transonic nozzle reduces energy loss and improved the energy efficiency of VT; disadvantages of the transonic nozzle are, encapsulated by the advantages. Bazgir et al.^12^ proposed a novel work on counter flow RHVT with 3D numerical value; used fins inside the cold tube at study state condition; geometry of fins with triangle square, rectangle, circle parallelogram, and trapezium, etc. Summarized results were established on the COP, isentropic efficiency, the temperature difference was recorded at maximum with parallelogram and minimum with rectangle fins. Bazgir et al.^13^ explained on temperature detachment was explained using different turbulence models of a straight tube with the counter flow of RHVT; RNG k–ϵ model with respective to FMV; The results were explained 20 divergence is the optimal and critical length as 166 mm temperature elimination with increased with the number of nozzles. Cartlidge et al.^14^ investigated with divergent vortex tube with an inlet pressure of 3–7 bar, with a mass flow rate range of 0.05–0.14 kg s^−1^. The half-angle of the hot tube was 3°; with six nozzles, the L/D 20; half angle conical splitter 45°. Liang et al.^15^ proposed an energy separation process on RHVT by the acoustic signal characteristics. Air as the inlet used at the inlet; nozzle numbers, and CMF were observed; maximum sound process level and COP with five nozzles compared with four and six nozzles on vortex tube was done. Nadernabhani^16^ explained that the comparison between cooling the hot tube with water and recorded flow and thermal patterns design used with the 3D study on airflow inside RHVT; Bazgir et al.^17^ explained the maximum and minimum temp difference and efficiencies were recorded at Nozzle number four at L/D = 47.5, and N = 1 at L/D = 42.5 respectively. Bazgir et al.^18^ investigated the flow field and temperature separation of VT with straight and five angles of convergent like 5°, 8°, 1°, 1.5° and 2°. The thermal relevant performance for all quantitative streamlines was carried out. Liang et al.^19^ carried out an investigation on nozzle number, cold cone angle, materials, and structural parameters of the vortex tube. They compared cold cone angle, vortex tube materials, inlet presser, and the number of nozzles. The vortex tube with six nozzles having a cone angle of two degrees yielded high efficiency (Supplementary Information).

Mirjalilia et al.^20^ investigated the 2D numerical technique on RHVT with CMF equal to 0.44; and the X/L = 0.05, 0.5, and 0.9-time steps. The dispensation of radial, axial, and tangential velocities with stagnation pressure and temperature were examined and reported on the Vortex cold outlet with no decomposition. Bazgir et al.^21^ explained the simulation of the desired separation in commercial vortex tube with hydrocarbons of surrogate for air; a mixture of 21% of cyclohexane and 79% n-pentane was chosen to evaluate feed conditions on vortex tube separation phenomenon. The fine separation was observed. This study suggests that the operation of the commercial vortex tube used resulted in mixing, introducing other processes that reduce separation compared to equilibrium ignition. Bazgir et al.^22^ proposed the analysis to enhance the performance of RHVT with a reduction in the temperature of cold air, isentropic efficiency, and COP of converging and diverging hot tube-were recorded. Two important parameters namely the length of the hot pipe, and the number of nozzles were considered for the applications of cooling and insulation, further they examined to determine how the VT process was affected by the various geometric configurations on the cooled tube. The all above factors were improved with cooled RHVT compared to un-cooled tubes. Bazgir et al.^23^ proposed in their investigation, computational fluid dynamics (CFD) analysis for the flow field, temperature field, and pressure field and optimize the VT parameters to achieve a specific set of desired output to meet the application requirements. Guo et al.^24^ proposed the basic distribution of deterministic frequencies and the relationship between frequency and energy separation, processing frequency characteristics were analyzed in vortex tubes with a diameter of 30 mm. An increase in energy vortex frequency gives a higher power transfer capability, which leads to higher performance. Based on these observations, the patterns of large-scale velocities and energy distribution of the vortex tube were determined. Bazgir et al.^25^ investigated obtaining the minimum cooling outlet temperature by changing the injection nozzle angle. In addition, the effect of pressure on the vortex tube chamber and its relationship to the cold exit temperature and the optimal nozzle passage was selected. Finally, some of the results of this report are supported by available experimental data. A comparison shows a fair deal. Bazgir et al.^26^ proposed the uniform and non-uniform hot tubes of convergent, divergent angles. The different divergent angles of 1°, 2°, 3°, 4° and 6° with two divergent angles of 1° and 2° were adjusted in hot tubes used to study the Isentropic efficiency and COP. There are several factors in this study such as inlet nozzle angle, inlet pressure, mass flow, and the number of inlet nozzles as well as the effects of different types of intake gas have been analyzed in detail to maximize turbine cooling efficiency (straight).

Abdelghany et al.^27^ explained the performance of the tube which is deeply influenced by the vortex tube L/D proportion and when it operated at various cold mass fractions. The maximum COP developed at cold mass fraction was found to be 0.64. Bramo et al.^28^ have observed in their research by CFD analysis the L/D ratio of the hot tube of 8, 9, 10.5, 20.2, 30.7, and 35 with 6 straight nozzles. Zin et al.^29^ presented the effect of the L/D ratio on fluid flow inside the RHVT using CFD simulation and evaluated experimental results. Nezhad et al.^30^ presented three dimensional CFD model to study the variation of flow and heat transfer in the vortex tube. Rattanongphisat et al.^31^ studied using the standard k–ϵ, 3-D numerical model predictions to simulate the physical performance of the flow such as pressure and temperature inside the Vortex tube. El May et al.^32^ presented a three-dimensional numerical model of RHVT using the CFD code (Fluent) to study the effect of the “cold end diameter” in the energy separation mechanism inside the vortex tube. Behera et al.^33^ presented their experiments on the number of nozzles and profiles, swirl, axial and radial velocity, also secondary zone circulation, and cross verified by CFD simulation. Baghdad et al.^34^ investigated through the 3D CFD domain on energy separation process and flow experience inside a vortex tube. Dutta et al.^35^ presented the CFD model which was used to simulate the phenomenon of energy separation in gaseous air at cryogenic temperature.

### Material importance on the performance of vortex tube

Several studies have been conducted to investigate the effect of using different materials on the performance of vortex tubes. Stainless steel is commonly used in vortex tubes; however, other materials were also used. These materials can be divided into two general classifications: metal and plastic materials. Metal materials include steel, copper, aluminum, alloys, etc., and plastic materials include Perspex, capralon, polystyrene, and others. According to Parulekar^36^, the internal roughness of the pipe also affects its performance: any internal roughness will reduce the efficiency of the system (due to temperature differences) by up to 20%. Saidi and Yazdi^37^ found that steel pipe has a greater temperature difference (T_h_–T_c_) than PVC pipe. They concluded that the use of materials with soft surfaces and low thermal conductivity leads to the effectiveness of the second law. Singh^38^ proposed the performance of Perspex pipe is normally greater than that of brass tube. The overall lower efficiency of the brass tube may be due to its better conductivity than the Perspex tube. Azarov^39^ developed various cast vortex tubes, such as stainless steel, copper, aluminum, aluminum alloy, capralon, textolite, etc. All researchers culminate that the use of smooth-edged materials with low thermal conductivity, and the use of insulated pipes reduce energy loss to the environment, resulting in better insulation and better performance.

Eiamsa-ard et al.^40^ presented the effects of the cooling of a hot tube on the temperature reduction of the cold air and the cooling efficiency of the counter-flow RHVT investigated experimentally. The hot tube material like Acrylic and Copper were used. By covering hot tubes with cooling water jackets. In this connection, the cooling of the hot tubes recorded more cooling efficiency than without cooling hot tubes. Hamdan et al.^41^ proposed using stainless steel material made VT with six numbers of nozzles with orientation and symmetric/asymmetry arrangements on maximum energy separation. It was observed that inlet pressure and vortex stopper location affect the performance of the VT.

Devade et al.^42^ proposed the effect of orifice diameter on the performance of the tube. Four different conical valves of angles of 30°, 45°, 60°, 90° are used on the hot end. By the results, it concluded that the converging type of vortex tube has proved to be promising as far as the optimization of cold mass fraction and lower cold end temperatures. It has satisfactorily produced a low temperature of about 5 °C and cold mass fractions of the order of 0.9 with COP as high as 0.202 were noticed. Saidi et al.^43^ explained in an experimental report on geometrical parameters of VT and the effect of inlet gas, type of gas effect on cold temperature, and efficiency was discussed. Yilmaz et al.^44^ investigated the VT design criteria, and types and reported on VT’s experimental and theoretical results.

## Problem statement

**Figure 1:**
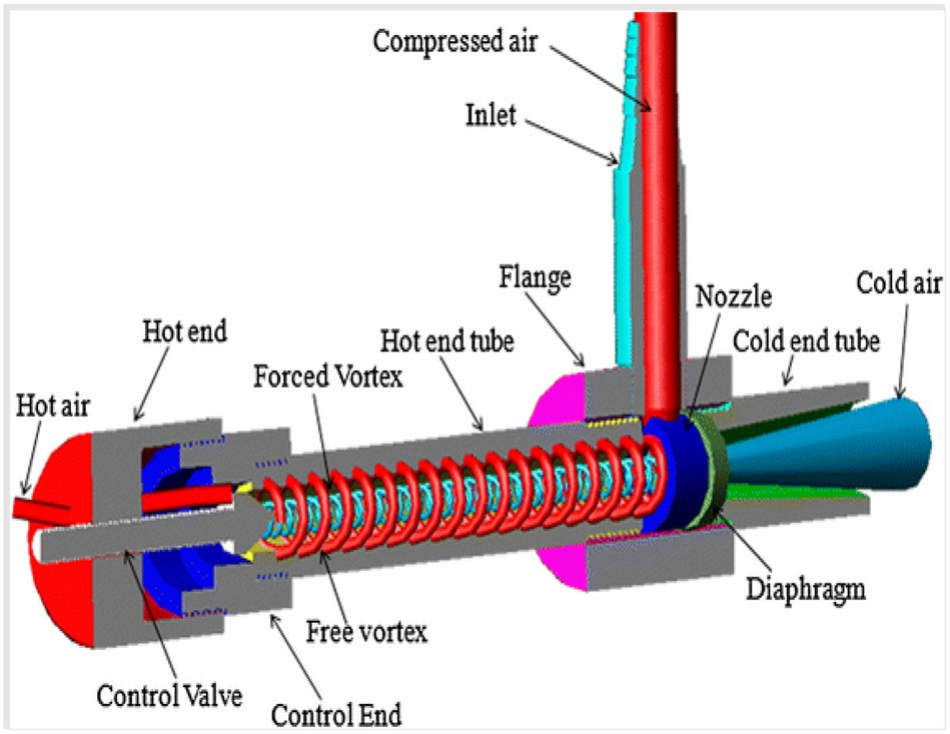
Vortex tube sectional view.

**Figure 2:**
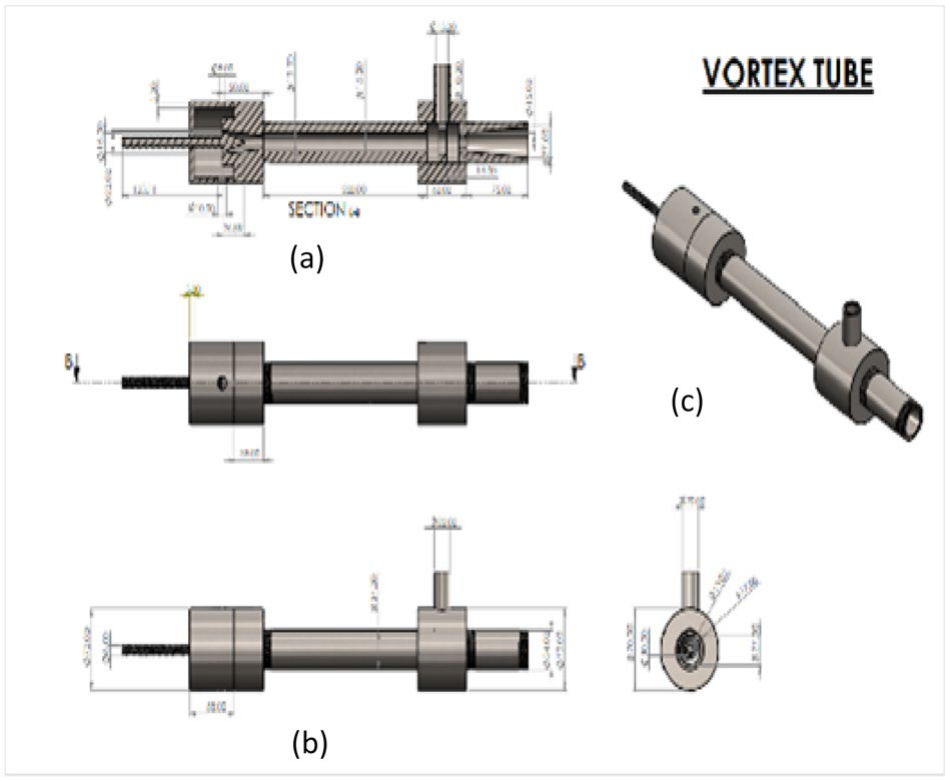
Vortex tube; (**a**) sectional view; (**b**) orthographic view; (**c**) 3D view.

The present work was carried out with different materials of vortex tubes considering one of the geometrical parameter’s lengths to diameter ratio of the hot tube (L/D) as 40 by using the constant inner diameter of the hot tube as 15 mm, the pressurized air was passed at the inlet from 2 to 12 bars through the nozzle as shown in Fig. 1. The sectional view along with the internal parts like Inlet, Tangential nozzle, Diaphragm (orifice), Flange (main chamber), hot tube, Hot end, control valve, and cold end tube are given. From Fig. 2 (a) sectional view, (b) orthographic view, and (c) 3-D vortex tube is clearly shown. The complete simulation was done by Ansys Fluent R-19 software. The simulation of a vortex tube with eight different materials was presented.

### Design and construction details

Materials used in simulation likewise copper, brass, bronze, stainless steel, PVC, CPVC, acrylic nylon, etc.

Length/diameter of hot tubes (L/D) = 600/15 = 40.

Nozzle diameter D_n_ = 10 mm.

Orifice diameter d_c_ = 8 mm.

Inlet pressure = 2–12 bar.

Conical needle angle = 35°.

The flow is analyzed by using CFD analysis Ansys Fluent R19.

### Experimental formula

- Cold mass fraction:
  1 $$\mu = \frac{({T}_{h} - {T}_{i})}{({T}_{h} - {T}_{c}) }$$
- The cooling temperature difference (∆T_c_) and hot temperature difference (∆T_h_) are expressed as follows:
  2 $$\Delta \text{T}_\text{c} = \text{T}_\text{i} -\text{ T}_\text{c}$$
  3 $$\Delta \text{T}_\text{h} = \text{T}_\text{h} -\text{ T}_\text{i}$$
- The performance of the vortex tube is determined by the variation in the center for the heating effect and for the cooling effect. Deduct Eq. (2) from Eq. (3) gives the performance of the vortex tube equation as follows Eq. (4):
  4 $$\Delta \text{T} = \text{T}_\text{h} -\text{ T}_\text{c}$$
- 5 $$\mathrm{Area}\, \mathrm{of} \, \mathrm{the} \,\mathrm{inlet}:(\text{A})= \pi /4\times \text{d}^{2}$$
- 6 $$\mathrm{Cold} \,\mathrm{mass} \,\mathrm{flow} \,\mathrm{rate }( \dot{m}_{c} ) = \rho \times \text{A}\times \text{V} \,(\text{kg} \,\text{s}^{-1})$$
  where A = area of the inlet − (m^2^).
  d = diameter of inlet (m).
  V = velocity of the air (m s^−1^).

(f) 7 $$\Delta {\text{T}^{\prime}}_{{\text{c}}} = {\text{T}}_{{\text{i}}} \left[ {1 - \left( {\frac{{{\text{P}}_{{\text{a}}} }}{{{\text{P}}_{{\text{i}}} }}} \right)^{{\frac{{\gamma - 1}}{\gamma }}} } \right]$$
(g) Relative temperature drop is
  8 $$\text{T}_\mathrm{rel }= \Delta \text{T}_\text{c}/\Delta \text{T}_\text{c}{^{\prime}}$$
(h) The adiabatic efficiency of the vortex tube is given as
  9 $$\eta _\text{ab} = \mu \Delta \text{T}_\text{rel}$$
(i) 10 $$\mathrm{Coefficient} \, \mathrm{of} \, \mathrm{performance} \, \mathrm{ of} \, \mathrm{the} \, \mathrm{Vortex} \, \mathrm{Tube}.$$
(j) The coefficient performance of the vortex tube is the ratio of the refrigeration effect to the energy required to provide compressed air at the inlet (work done by the compressor).
  $${\rm{COP}} = \frac{{{\rm{Cooling~effect}}}}{{{\rm{work~input}}}}\quad {\rm{COP}} = \frac{{\mu ~\left( {\Delta T} \right)~\eta c~}}{{~\left[ {Ti\left\lfloor {{{\left( {\frac{{Pa}}{{Pi}}} \right)}^{\frac{{\gamma - 1}}{\gamma }}} - 1} \right\rfloor } \right]}}$$

Where:

µ = Cold mass fraction.

∆T = T_h_ − T_c_ (temperature difference).

$\eta {\text{c}}$ = Efficiency of the compressor.

Pi = inlet pressure.

P_a_ = Atmospheric pressure.

γ = Specific heat ratio (C_p_ C_v_^−1^).

T_i_ = Inlet temperature.

## CFD analysis of different materials

### Pressure at 2 bar

**Figure 3:**
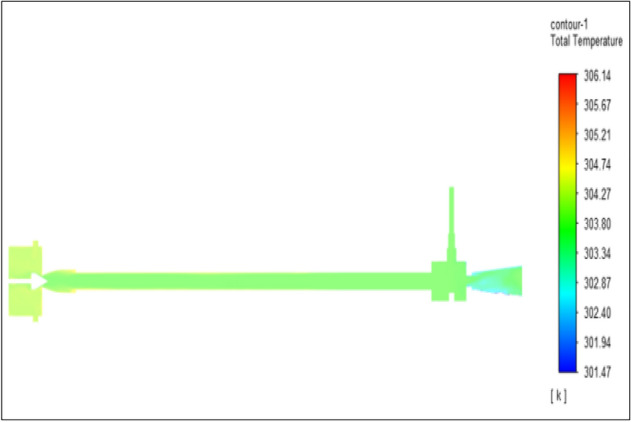
Stainless steel at 2 bar.

Figure 3 related to stainless steel vortex tube with an inlet pressure of 2 bar. The inlet temperature of the VT is 303 K. The hot air temperature was obtained at the hot end as 306.12 K. Similarly at cold end side obtained a value of 301.47 K. The maximum temperature difference was found to be 4.67 K.

**Figure 4:**
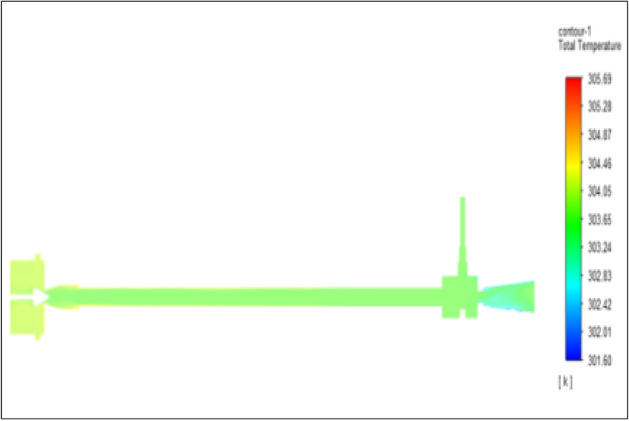
Brass at 2 bar.

Figure 4 related to the brass vortex tube with an inlet pressure of 2 bar. The inlet temperature of the VT is 303 K. The hot air temperature was obtained at the hot end as 305.69 K, and at the cold end side obtained a value of 301.6 K. The maximum temperature difference was found to be 4.08 K.

**Figure 5:**
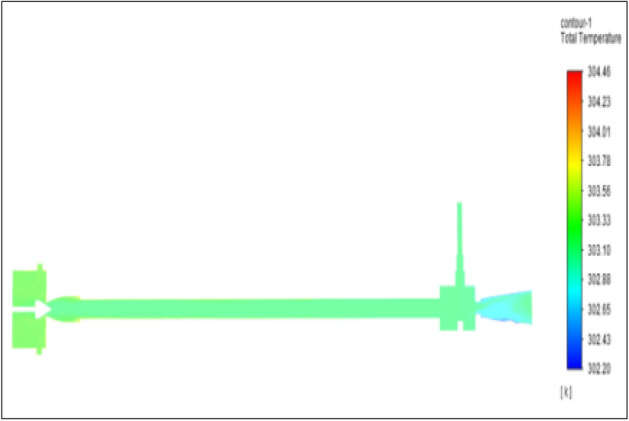
PVC at 2 bar.

Figure 5 represented to PVC vortex tube with an inlet pressure of 2 bar. The inlet temperature of the VT is 303 K. The hot air temperature was obtained at the hot end as 304.46 K, and at the cold end side obtained a value of 302.20 K. The maximum temperature difference was obtained as 2.26 K.

**Figure 6:**
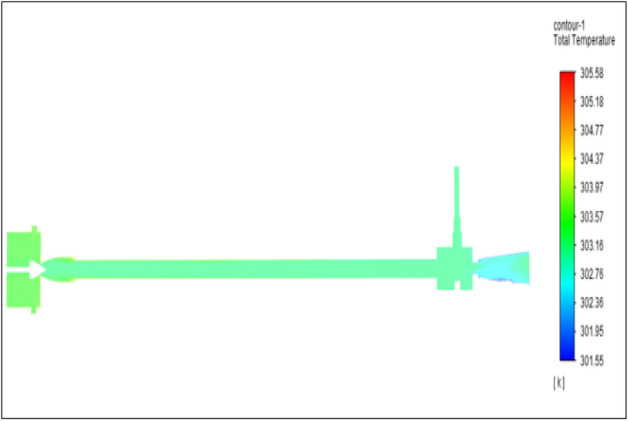
CPVC at 2 bar.

Figure 6 represents to CPVC vortex tube with an inlet pressure of 2 bars. The inlet temperature of the VT is 303 K. The hot air temperature was obtained at the hot end as 305.08 K, and at the cold end side obtained a value of 301.55 K. The maximum temperature difference was obtained a 3.54 K.

**Figure 7:**
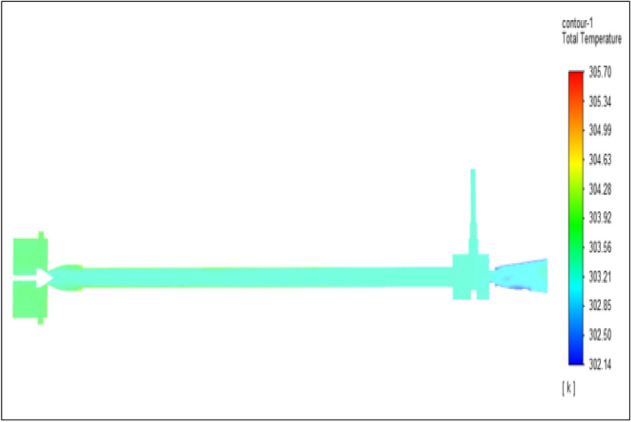
Acrylic at 2 bar.

Figure 7 represents to acraulic vortex tube with an inlet pressure of 2 bars. The inlet temperature of the VT is 303 K. The hot air temperature was obtained at the hot end as 305.69 K, and  at the cold end side obtained a value of 302.14 K. The maximum temperature difference was obtained as 3.55 K.

**Figure 8:**
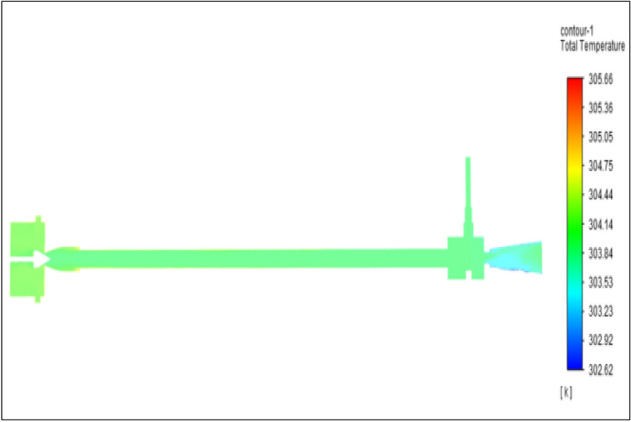
Nylon at 2 bar.

Figure 8 represents to nylon vortex tube with an inlet pressure of 2 bars. The inlet temperature of the VT is 303 K. The hot air temperature was obtained at the hot end as 305.65 K, and at the cold end side obtained a value of 302.62 K. The maximum temperature difference was obtained as 3.03 K.

**Figure 9:**
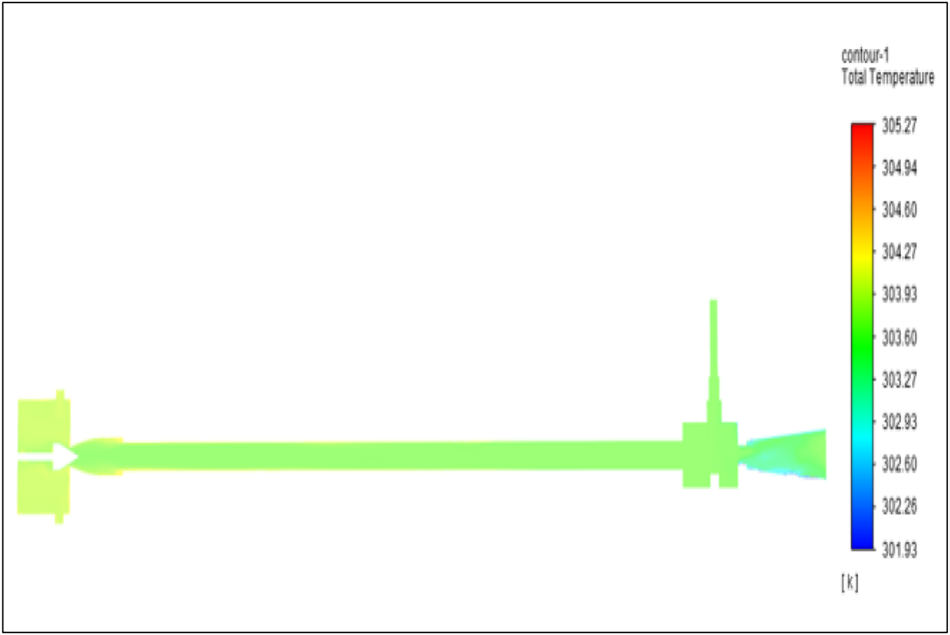
Bronze at 2 bar.

Figure 9 represents to bronze vortex tube with an inlet pressure of 2 bars. The inlet temperature of the VT is 303 K. The hot air temperature was obtained at the hot end as 305.65 K, at the cold end side obtained a value of 301.93 K. The maximum temperature difference was found to be 3.35 K.

**Figure 10:**
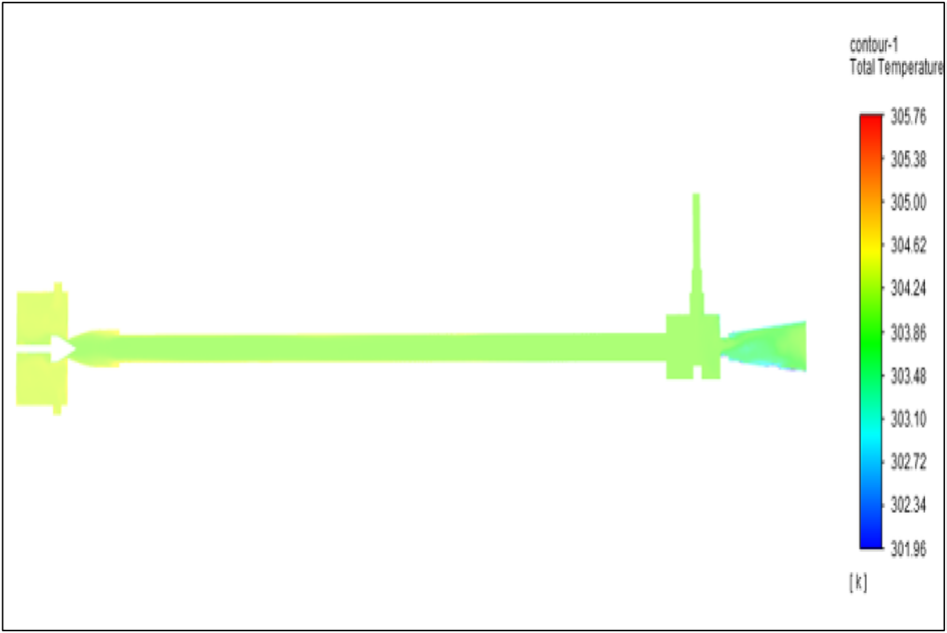
Copper at 2 bar.

Figure 10 represents to copper vortex tube with an inlet pressure of 2 bars. The inlet temperature of the VT is 303 K. The hot air temperature was obtained at the hot end as 305.75 K and at the cold end side obtained a value of 301.96 K. The maximum temperature difference was found to be 3.78 K.

### Pressure at 4 bar

**Figure 11:**
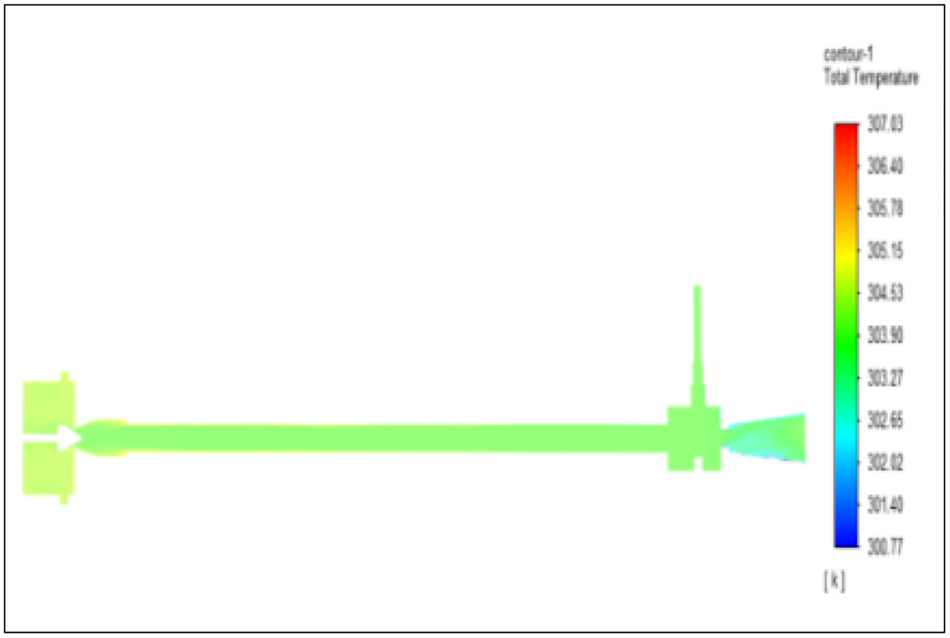
Stainless steel at 4 bar.

Figure 11 related to stainless steel vortex tube with an inlet pressure of 4 bars. The inlet temperature of the VT is 303 K. The hot air temperature was obtained at hot the end as 307.03 K. Similarly at cold end side obtained a value of 300.77 K. The Maximum temperature difference was found to be 6.25 K.

**Figure 12:**
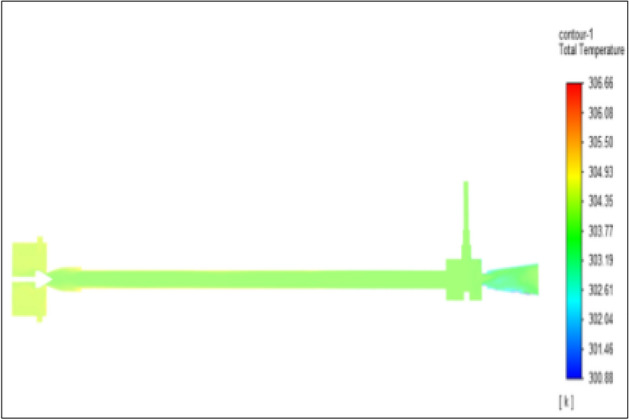
Brass at 4 bar.

Figure 12 related to a brass vortex tube with an inlet pressure of 4 bars. The inlet temperature of the VT is 303 K. The hot air temperature was obtained at the hot end as 306.67 K. Similarly, the cold end side obtained a value of 300.88 K. The maximum temperature difference was found to be 5.79 K.

**Figure 13:**
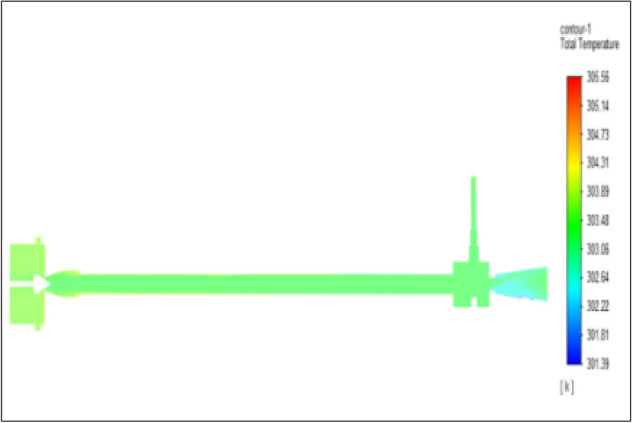
PVC at 4 bar.

Figure 13 represents to PVC Vortex tube with an inlet pressure of 4 bars. The inlet temperature of the VT is 303 K. The hot air temperature was obtained at the hot end as 305.56 K and at the cold end side obtained a value of 301.39 K. The maximum temperature difference was found to be 4.18 K.

**Figure 14:**
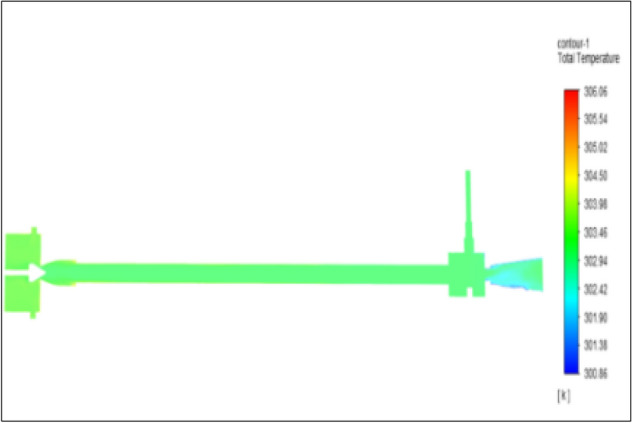
CPVC at 4 bar.

Figure 14 represents to CPVC vortex tube with an inlet pressure of 4 bars. The inlet temperature of the VT is 303 K. The hot air temperature was obtained at the hot end as 306.06 K. Similarly, the cold end side obtained a value of 300.86 K. The maximum temperature difference was found to be 5.21 K.

**Figure 15:**
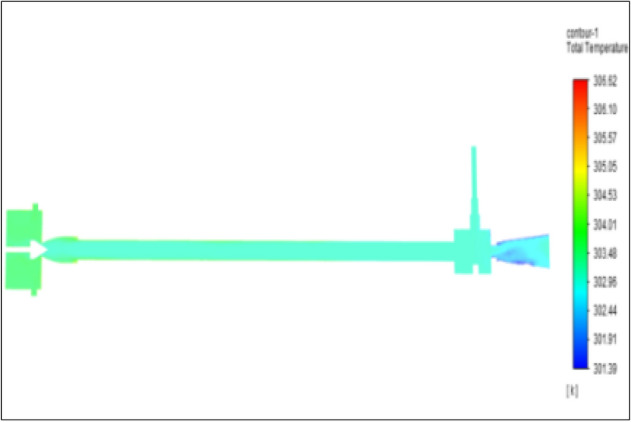
Acrylic at 4 bar.

Figure 15 represents to acraulic vortex tube with an inlet pressure of 4 bars. The inlet temperature of the VT is 303 K. The hot air temperature was obtained at the hot end as 306.61 K. Similarly at cold end side obtained a value of 301.39 K. The maximum temperature difference was found to be at 5.23 K.

**Figure 16:**
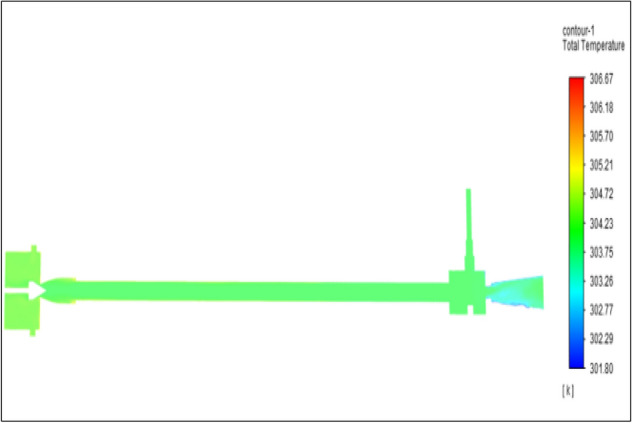
Nylon at 4 bar.

Figure 16 represents to nylon vortex tube with an inlet pressure of 4 bars. The inlet temperature of the VT is 303 K. The hot air temperature was obtained at the hot end as 306.66 K. Similarly at cold end side obtained a value of 301.8 K. The maximum temperature difference was found to be 4.86 K.

**Figure 17:**
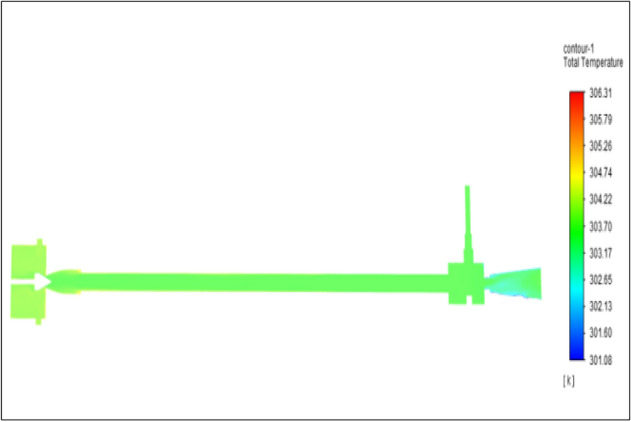
Bronze at 4 bar.

Figure 17 represents to bronze vortex tube with an inlet pressure of 4 bars. The inlet temperature of the VT is 303 K. The hot air temperature was obtained at the hot end as 305.31 K. Similarly at cold end side obtained a value of 301.08 K. The maximum temperature difference was found to be 5.23 K.

**Figure 18:**
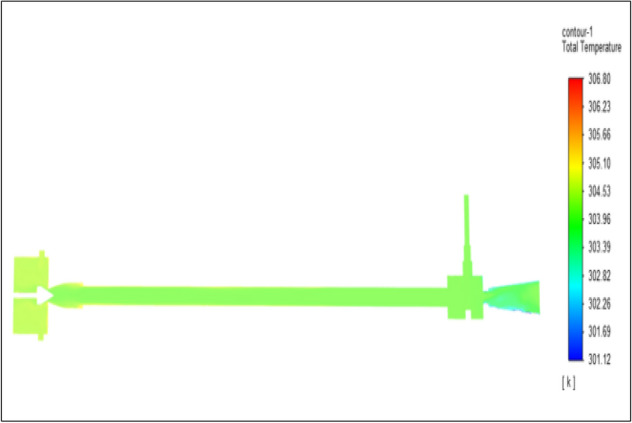
Copper at 4 bar.

Figure 18 represents to copper vortex tube with an inlet pressure of 4 bars. The inlet temperature of the VT is 303 K. The hot air temperature was obtained at the hot end as 306.79 K and at the cold end side obtained a value of 301.12 K. The maximum temperature difference was found to be 5.67 K.

### Pressure at 6 bar

**Figure 19:**
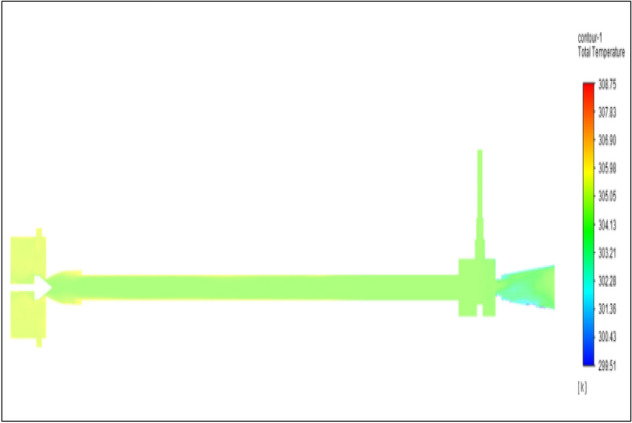
Stainless steel at 6 bar.

Figure 19 related to stainless steel vortex tube with an inlet pressure of 6 bars. The inlet temperature of the VT is 303 K. The hot air temperature was obtained at hot end as 307.89 K and  at the cold end side obtained a value of 300.11 K. The maximum temperature difference was noticed as 7.78 K.

**Figure 20:**
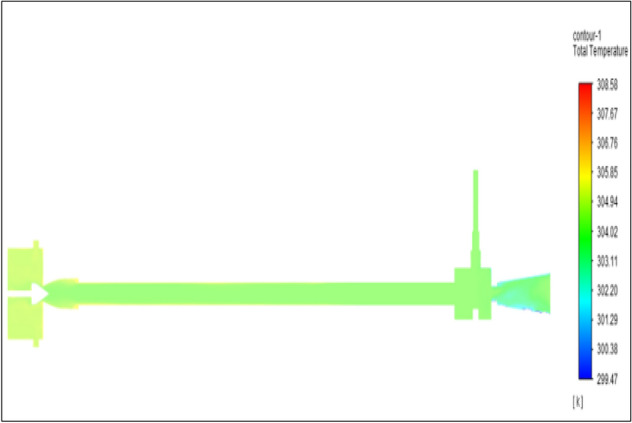
Brass at 6 bar.

Figure 20 has related to the brass vortex tube with an inlet pressure of 6 bars. The inlet temperature of the VT is 303 K. The hot air temperature was obtained at the hot end as 307.62 K at the cold end side obtained a value of 300.19 K. The maximum temperature difference was found to be 7.43 K.

**Figure 21:**
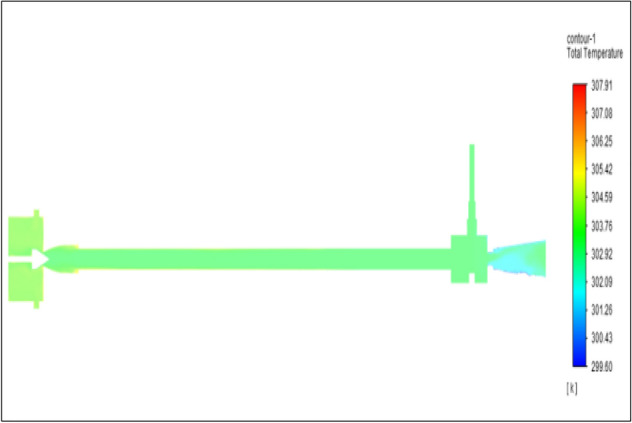
PVC at 6 bar.

Figure 21 has represented to PVC vortex tube with an inlet pressure of 6 bars. The inlet temperature of the VT is 303 K. The hot air temperature was obtained at the hot end as 306.04 K. Similarly at the cold end side obtained a value as 300.60 K. The maximum temperature difference was noticed as 6.03 K.

**Figure 22:**
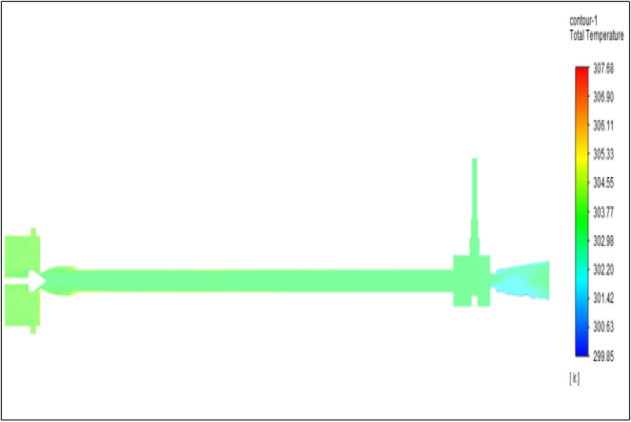
CPVC at 6 bar.

Figure 22 has represented to CPVC vortex tube with an inlet pressure of 6 bars. The inlet temperature of the VT is 303 K. The hot air temperature was obtained at the hot end as 307.02 K. Similarly at the cold end side obtained value was 300.19 K. The maximum temperature difference was found to be 6.82 K.

**Figure 23:**
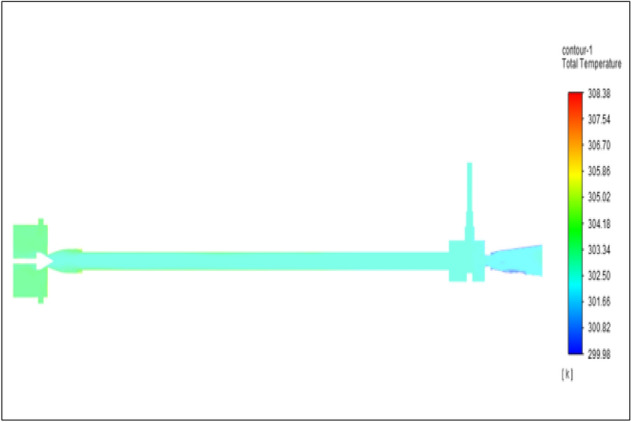
Acrylic at 6 bar.

Figure 23 has represented to acraulic vortex tube with an inlet pressure of 6 bars. The inlet temperature of the VT is 303 K. The hot air temperature was obtained at the hot end as 307.51 K. Similarly at cold the end side obtained a value of 300.67 K. The maximum temperature difference was noticed as 6.84 K.

**Figure 24:**
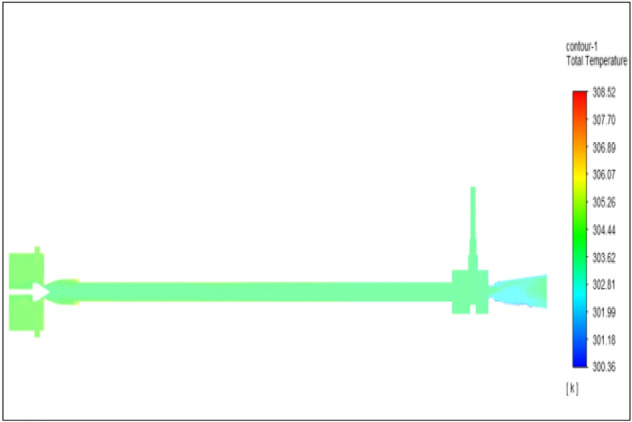
Nylon at 6 bar.

Figure 24 represented to nylon vortex tube with an inlet pressure of 6 bars. The inlet temperature of the VT is 303 K. The hot air temperature was obtained at the hot end as 307.61 K. Similarly at the cold end side obtained value was 301.05 K. The maximum temperature difference was found to be 6.56 K.

**Figure 25:**
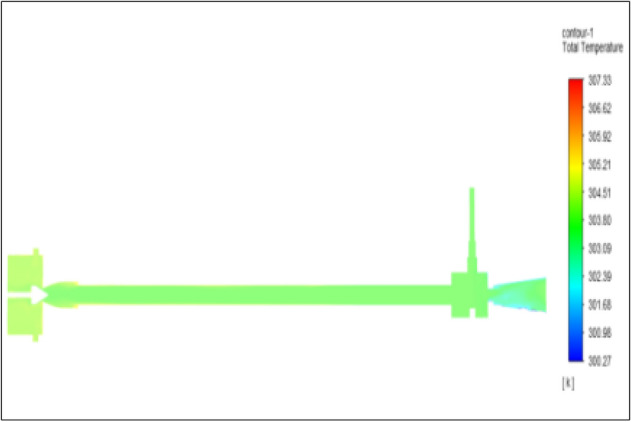
Bronze at 6 bar.

Figure 25 represented to bronze vortex tube with an inlet pressure of 6 bars. The inlet temperature of the VT is 303 K. The hot air temperature was obtained at the hot end as 307.33 K. Similarly at the cold end side obtained value of 300.27 K. The maximum temperature difference was noticed as 7.06 K.

**Figure 26:**
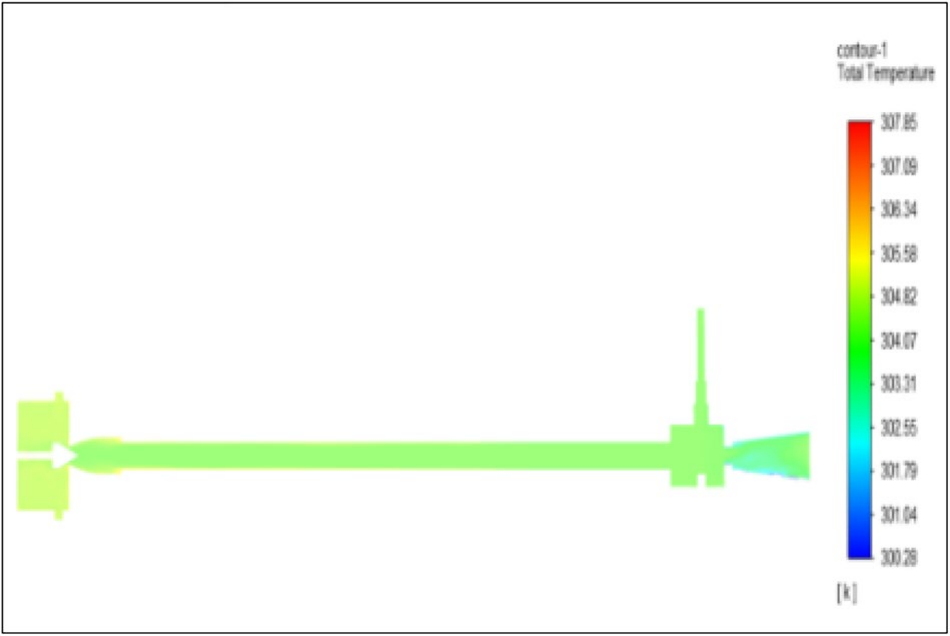
Copper at 6 bar.

Figure 26 represented to copper vortex tube with an inlet pressure of 6 bars. The inlet temperature of the VT is 303 K. The hot air temperature was obtained at the hot end as 307.84 K. Similarly at the cold end side obtained value of 300.28 K. The maximum temperature difference was found to be 7.56 K.

### Pressure at 8 bar

**Figure 27:**
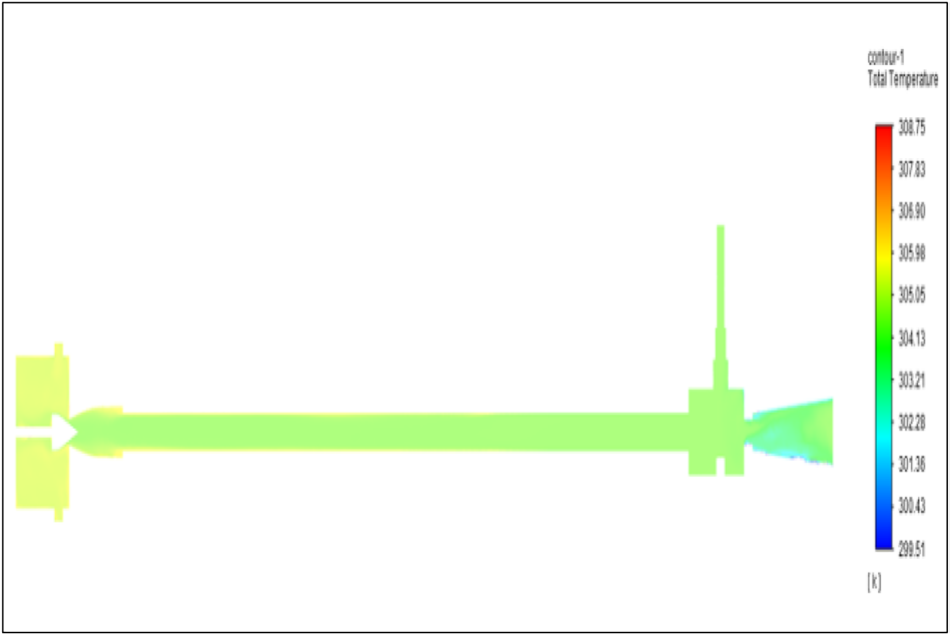
Stainless steel at 8 bar.

Figure 27 related to stainless steel vortex tube with an inlet pressure of 8 bars. The inlet temperature of the VT is 303 K. The hot air temperature was obtained at the hot end as 308.75 K. Similarly at the cold end side obtained value of 299.51 K. The maximum temperature difference was found to be 9.24 K.

**Figure 28:**
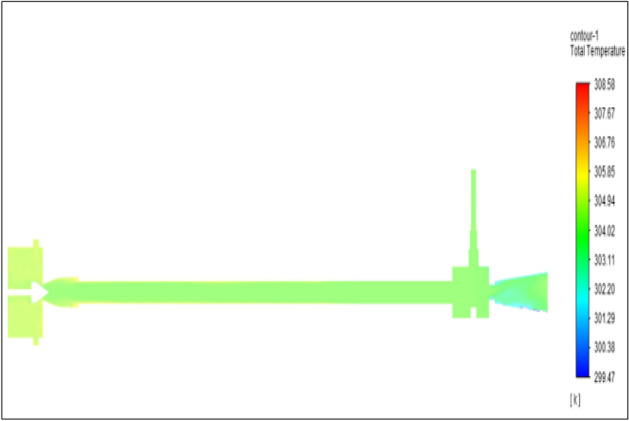
Brass at 8 bar.

Figure 28 related to a brass vortex tube with an inlet pressure of 8 bars. The inlet temperature of the VT is 303 K. The hot air temperature was obtained at the hot end as 308.58 K. Similarly at cold end side obtained a value of 299.47 K. The maximum temperature difference was found to be 9.11 K.

**Figure 29:**
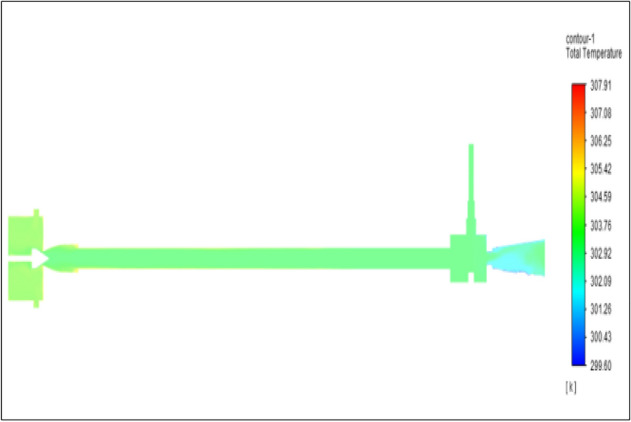
PVC at 8 bar.

Figure 29 represents to PVC vortex tube with inlet pressure of 8 bars. The inlet temperature of the VT is 303 K. The hot air temperature was obtained at hot end as 307.68 K. Similarly, the cold end side obtained a value of 299.85 K. The maximum temperature difference was observed at 7.83 K.

**Figure 30:**
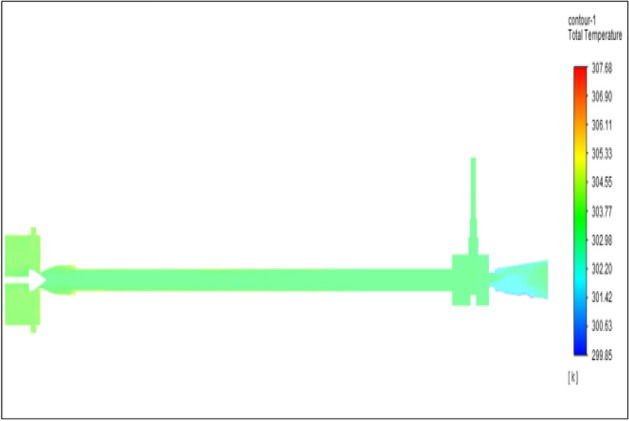
CPVC at 8 bar.

Figure 30 represents to CPVC vortex tube with inlet pressure at 8 bars. The inlet temperature of the VT is 303 K. The hot air temperature was obtained at the hot end as 307.91 K. Similarly at cold end side obtained a value of 299.60 K. The maximum temperature difference was observed as 8.31 K.

**Figure 31:**
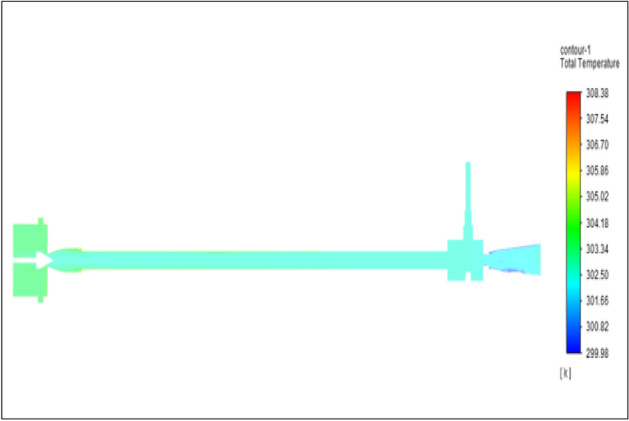
Acrylic at 8 bar.

Figure 31 represents to acraulic vortex tube with inlet pressure at 8 bars. The inlet temperature of the VT is 303 K. The hot air temperature was obtained at the hot end as 308.51 K, and at the cold end side obtained a value of 299.98 K. The maximum temperature difference was observed as 8.39 K.

**Figure 32:**
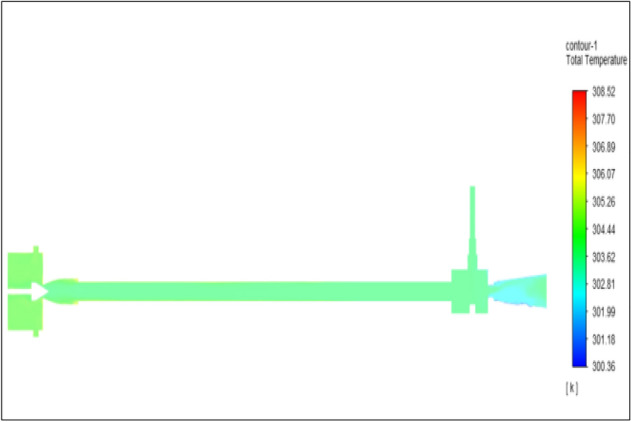
Nylon at 8 bar.

Figure 32 represents to nylon vortex tube with inlet pressure at 8 bars. The inlet temperature of the VT is 303 K. The hot air temperature was obtained at the hot end as 308.51 K, and at the cold end side obtained value as 300.36 K. The maximum temperature difference was observed as 8.15 K.

**Figure 33:**
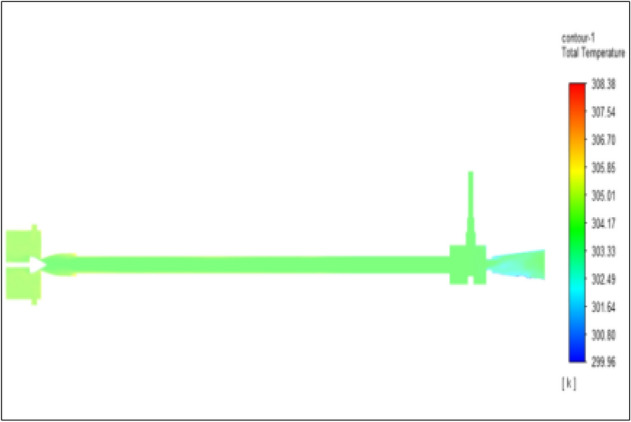
Bronze at 8 bar.

Figure 33 represents to bronze vortex tube with inlet pressure at 8 bars. The inlet temperature of the VT is 303 K. The hot air temperature was obtained at hot end as 308.38 K, at the cold end side obtained a value of 299.49 K. The maximum temperature difference was observed as 8.88 K.

**Figure 34:**
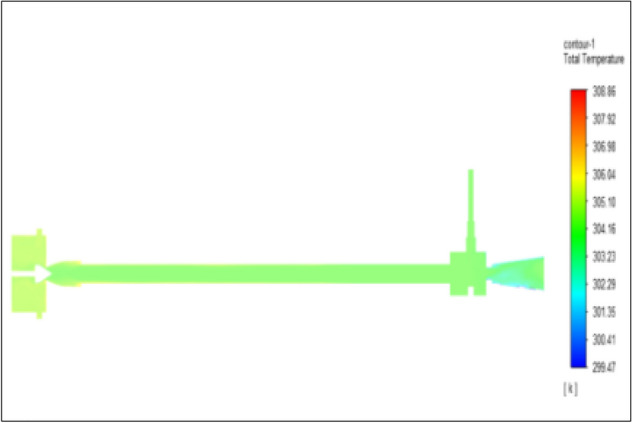
Copper at 8 bar.

Figure 34 represents to copper vortex tube with inlet pressure at 8 bars. The inlet temperature of the VT is 303 K. The hot air temperature was obtained at the hot end as 299.47 K, at the cold end side obtained value of 300.28 K. The maximum temperature difference was observed as 9.38 K.

### Pressure at 10 bar

**Figure 35:**
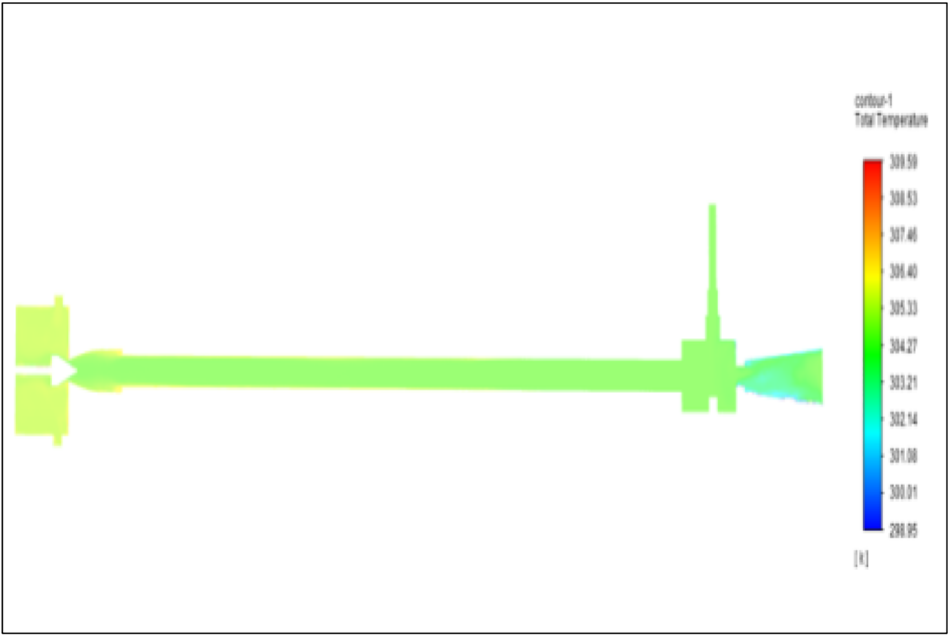
Stainless steel at 10 bar.

Figure 35 related to stainless steel vortex tube with an inlet pressure of 10 bars. The inlet temperature of the VT is 303 K. The hot air temperature was obtained at the hot end as 309.59 K. Similarly, the cold end side obtained a value of 298.95 K. The maximum temperature difference was noticed as 10.64 K.

**Figure 36:**
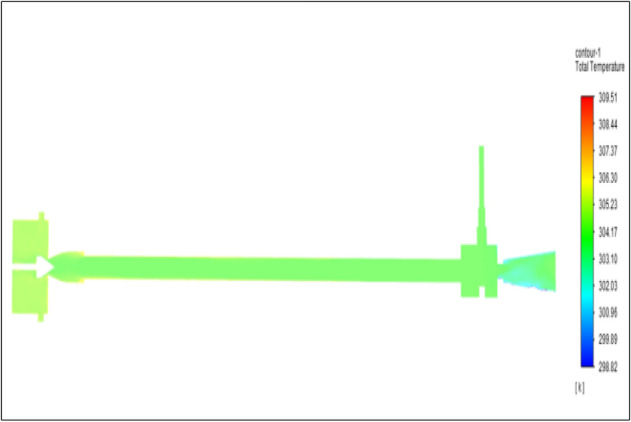
Brass at 10 bar.

Figure 36 related to a brass vortex tube with an inlet pressure of 10 bars. The inlet temperature of the VT is 303 K. The hot air temperature was obtained at the hot end as 309.51 K. Similarly at the cold end side obtained value was 298.82 K. The maximum temperature difference was noticed as 10.69 K.

**Figure 37:**
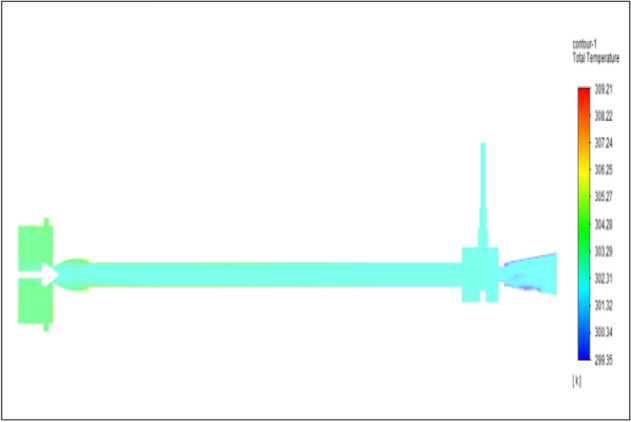
PVC at 10 bar.

Figure37 represents to PVC vortex tube with an inlet pressure of 10 bars. The inlet temperature of the VT is 303 K. The hot air temperature was obtained at hot end as 308.70 K. Similarly, the cold end side obtained a value of 299.17 K. The maximum temperature difference was noticed as 9.53 K.

**Figure 38:**
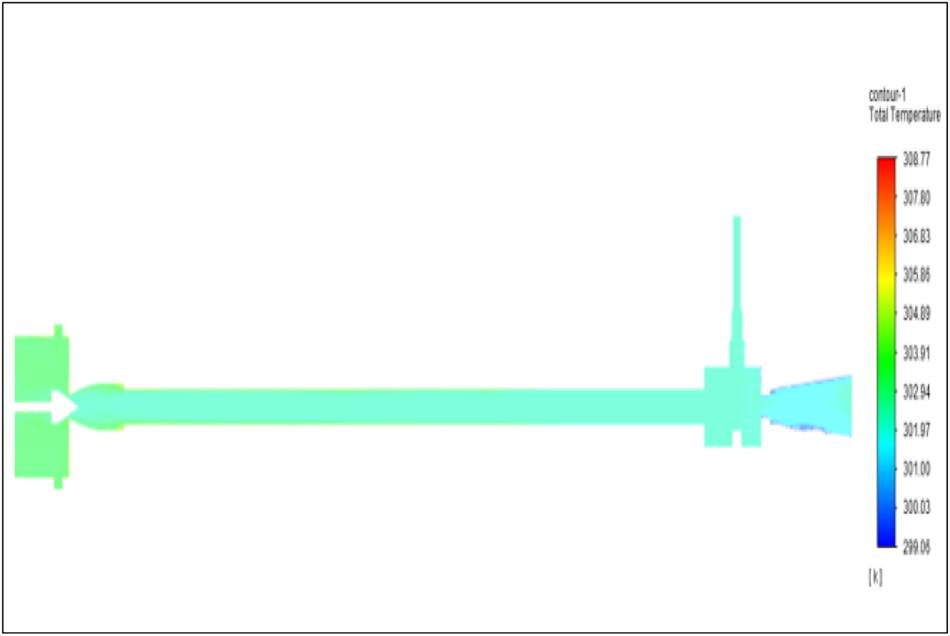
CPVC at 10 bar.

Figure 38 represents to CPVC vortex tube with an inlet pressure of 10 bars. The inlet temperature of the VT is 303 K. The hot air temperature was obtained at the hot end as 308.77. Similarly, at cold end side obtained a value of 299.06 K. The maximum temperature difference was noticed as 9.72 K.

**Figure 39:**
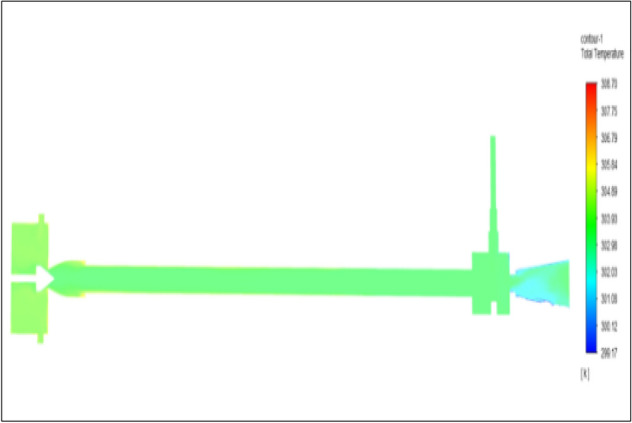
Acrylic at 10 bar.

Figure 39 represents to acraulic vortex tube with an inlet pressure of 10 bars. The inlet temperature of the VT is 303 K. The hot air temperature was obtained at the hot end as 309.20 K. Similarly, the cold end side obtained a value of 299.35 K. The maximum temperature difference was noticed as 9.86 K.

**Figure 40:**
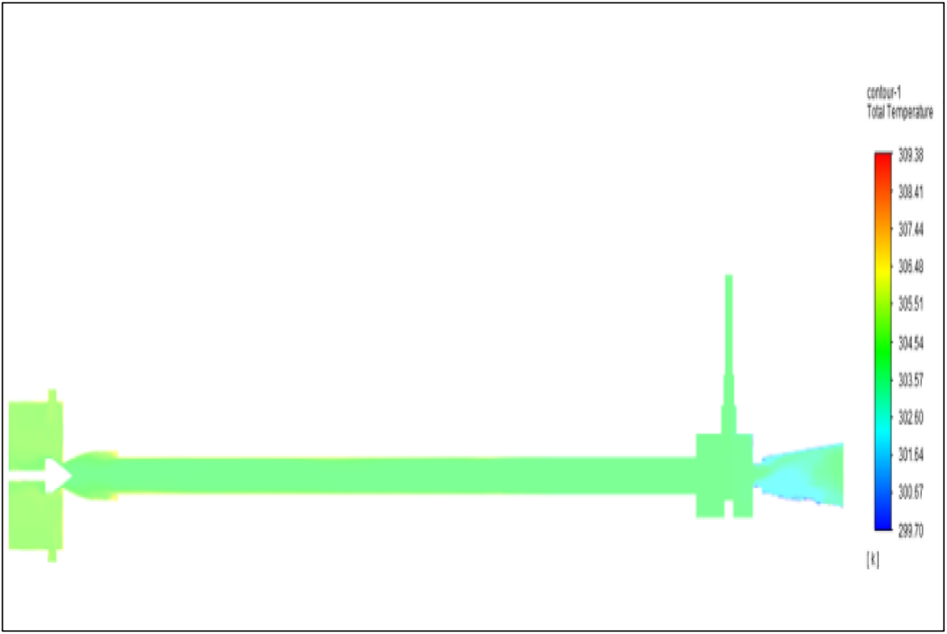
Nylon at 10 bar.

Figure 40 represents to nylon vortex tube with an inlet pressure of 10 bars. The inlet temperature of the VT is 303 K. The hot air temperature was obtained at the hot end as 309.37 K. Similarly, the cold end side obtained a value of 299.70 K. The maximum temperature difference was noticed as 9.67 K.

**Figure 41:**
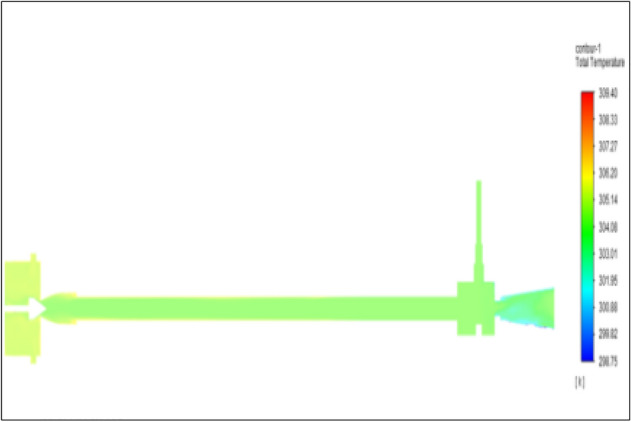
Bronze at 10 bar.

Figure 41 represents to bronze vortex tube with an inlet pressure of 10 bars. The inlet temperature of the VT is 303 K. The hot air temperature was obtained at the hot end as 309.40 K. Similarly at the cold end side obtained value was 298.75 K. The maximum temperature difference was noticed as 10.65 K.

**Figure 42:**
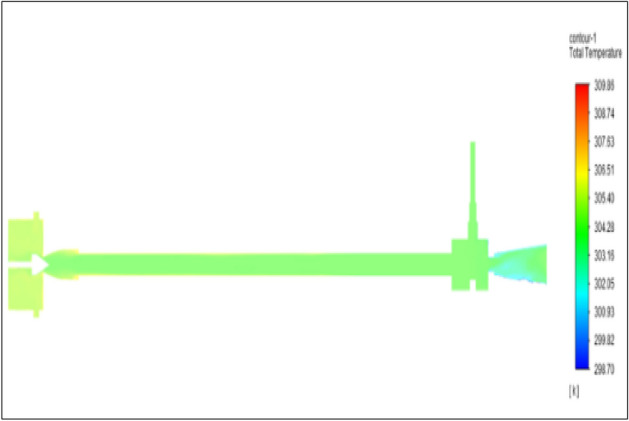
Copper at 10 bar.

Figure 42 represents to copper vortex tube with an inlet pressure of 10 bars. The inlet temperature of the VT is 303 K. The hot air temperature was obtained at hot end as 309.85 K. Similarly at cold end side obtained a value of 298.70 K. The maximum temperature difference was noticed as 11.15 K.

### Pressure at 12 bar

**Figure 43:**
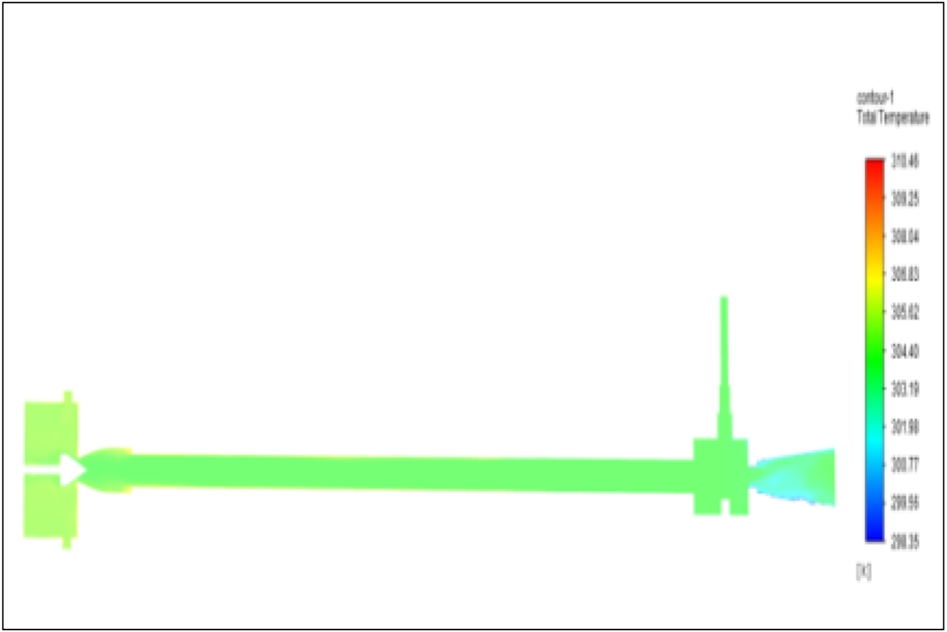
Stainless steel at 12 bar.

Figure 43 related to stainless steel vortex tube with an inlet pressure of 12 bars. The inlet temperature of the VT is 303 K. The hot air temperature was obtained at the hot end as 310.46 K. Similarly at the cold end side obtained value was 298.35 K. The maximum temperature difference was noticed as 12.11 K.

**Figure 44:**
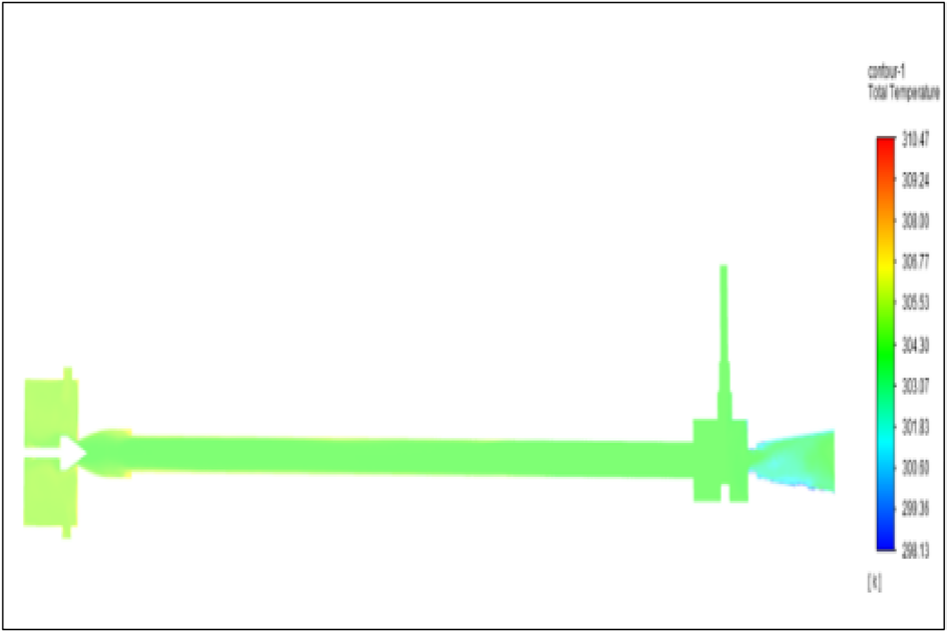
Brass at 12 bar.

Figure 44 related to a brass vortex tube with an inlet pressure of 12 bars. The inlet temperature of the VT is 303 K. The hot air temperature was obtained at the hot end as 310.47 K. Similarly at the cold end side obtained value was 298.13 K. The maximum temperature difference was observed as 12.34 K.

**Figure 45:**
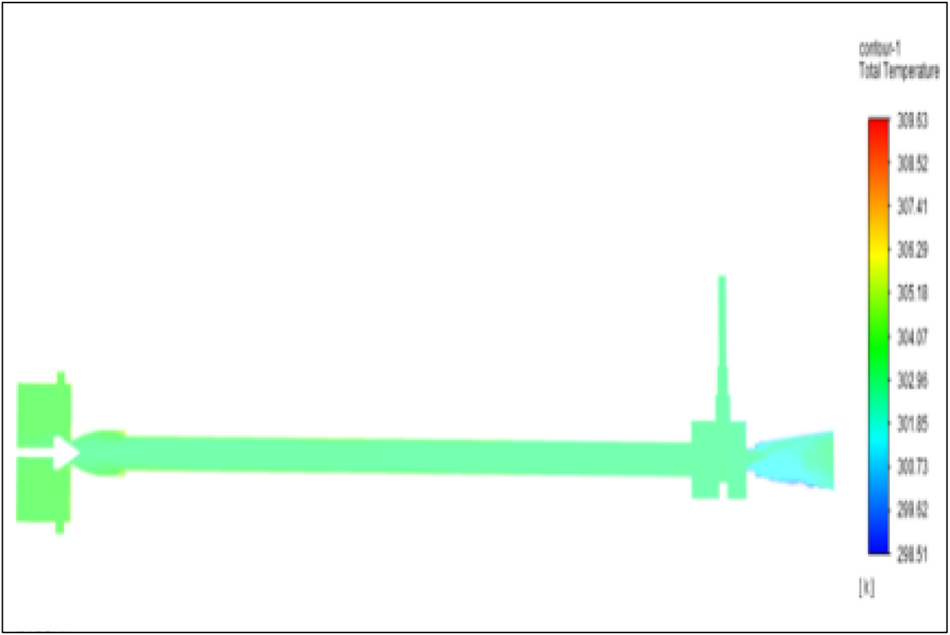
PVC at 12 bar.

Figure 45 represents to PVC vortex tube with an inlet pressure of 12 bars. The inlet temperature of the VT is 303 K. The hot air temperature was obtained at the hot end as 309.63 K. Similarly at the cold end side obtained a value of temperature as 298.51 K. The maximum temperature difference was noticed as 11.12 K.

**Figure 46:**
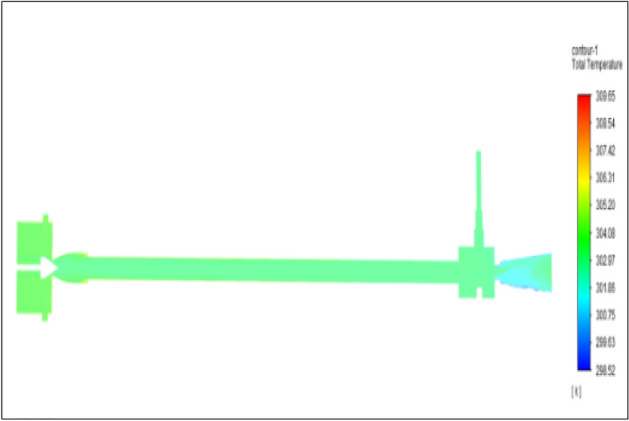
CPVC at 12 bar.

Figure 46 represents to CPVC vortex tube with an inlet pressure of 12 bars. The inlet temperature of the VT is 303 K. The hot air temperature was obtained at the hot end as 309.64. Similarly at cold end side obtained a value of 298.52 K. The maximum temperature was found to be 11.112 K.

**Figure 47:**
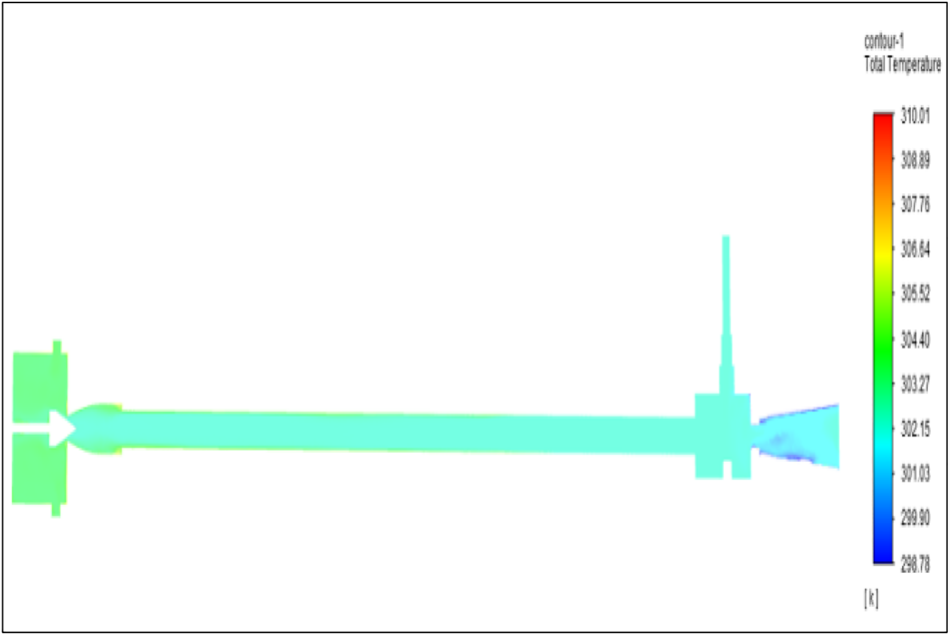
Acrylic at 12 bar.

Figure 47 represents to acraulic vortex tube with an inlet pressure of 12 bars. The inlet temperature of the VT is 303 K. The hot air temperature was obtained at the hot end as 310.02 K. Similarly at the cold end side obtained a value of temperature as 298.78 K. The maximum temperature difference was found to be 11.12 K.

**Figure 48:**
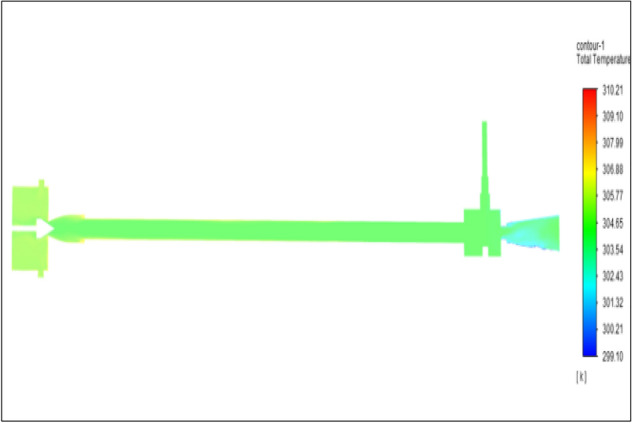
Nylon at 12 bar.

Figure 48 represents to nylon vortex tube with an inlet pressure of 12 bars. The inlet temperature of the VT is 303 K. The hot air temperature was obtained at the hot end as 310.22 K.

Similarly, at the cold end side obtained value was 299.10 K. The maximum temperature difference was found to be 11.1 K.

**Figure 49:**
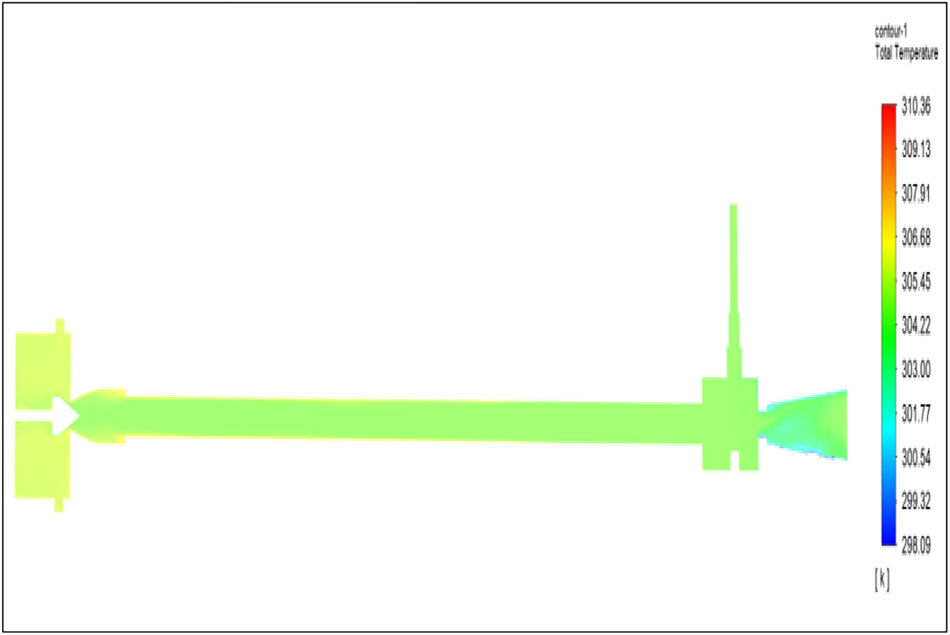
Bronze at 12 bar.

Figure 49 represents to bronze vortex tube with an inlet pressure of 12 bars. The inlet temperature of the VT is 303 K. The hot air temperature was obtained at the hot end as 310.36 K. Similarly, at the cold end side obtained temperature value as 298.09 K. The maximum temperature difference was found to be 12.27 K.

**Figure 50:**
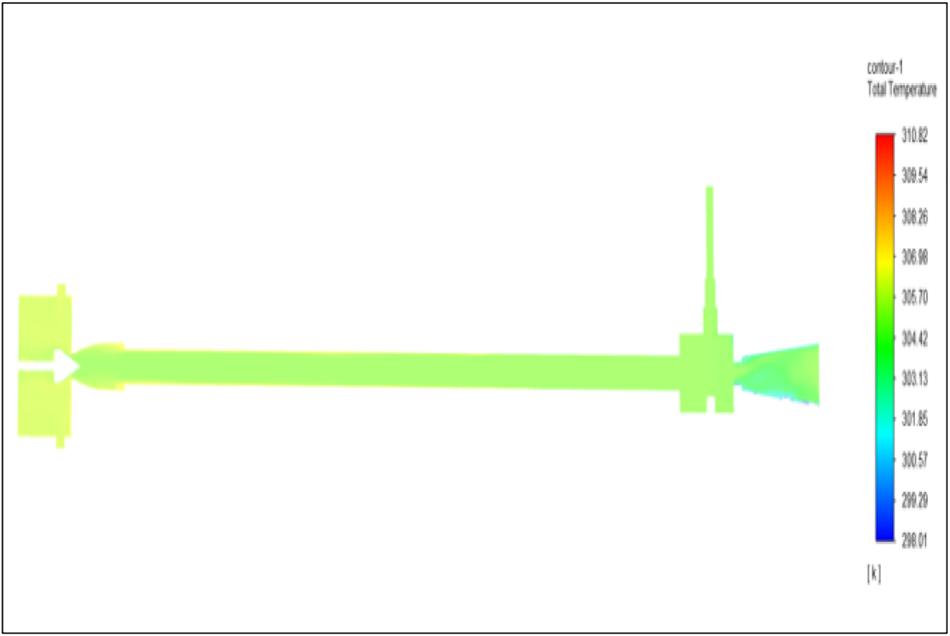
Copper at 12 bar.

Figure 50 represent to copper vortex tube with an inlet pressure of 12 bars. The inlet temperature of the VT is 303 K. The hot air temperature was obtained at the hot end as 310.81 K. Similarly, at cold end side obtained a temperature value of 298.01 K. The maximum temperature difference was recorded as 12.8 K.

## Pressure contours at 2–12 bar

**Figure 51:**
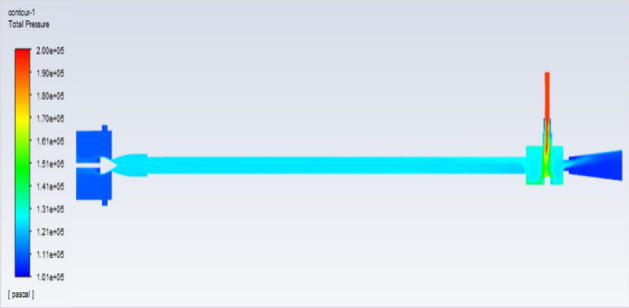
Pressure contours at 2 bar.

**Figure 52:**
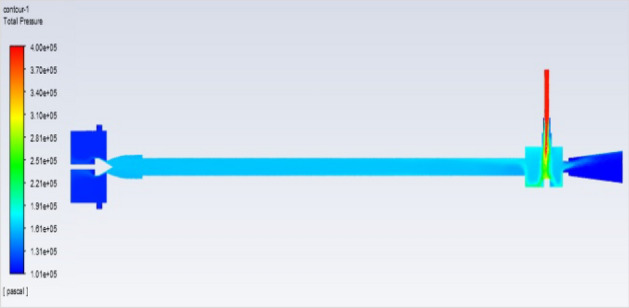
Pressure contours at 4 bar.

**Figure 53:**
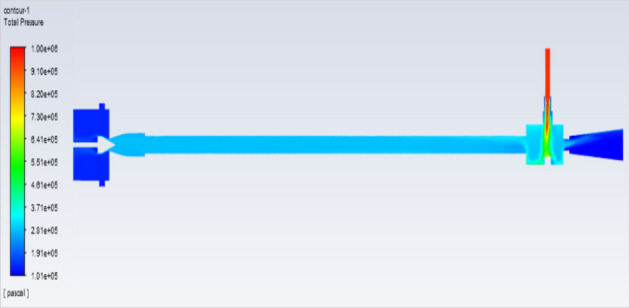
Pressure contours at 6 bar.

Figure 51 represents to vortex tube with an inlet pressure of 2 bars (2,000,000 Pa) at the outlet  and a minimum pressure of 101,325 Pa. Figure 52 represents to vortex tube with inlet with a maximum pressure of 4 bars (4,000,000 Pa) and at the outlet the vortex tube has a minimum pressure of 101,325 Pa. Figure 53 represents to vortex tube with an inlet pressure maximum of 6 bars (6,000,000 Pa) and at the outlet, the vortex tube has a minimum pressure of 101,325 Pa.

**Figure 54:**
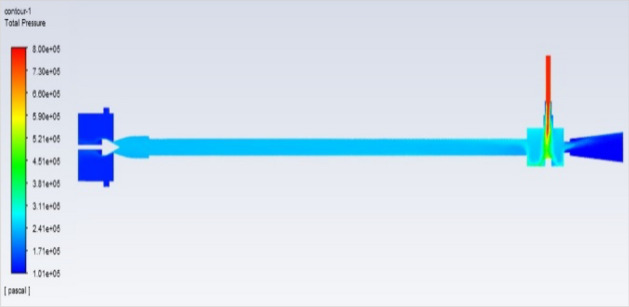
Pressure contours at 8 bar.

**Figure 55:**
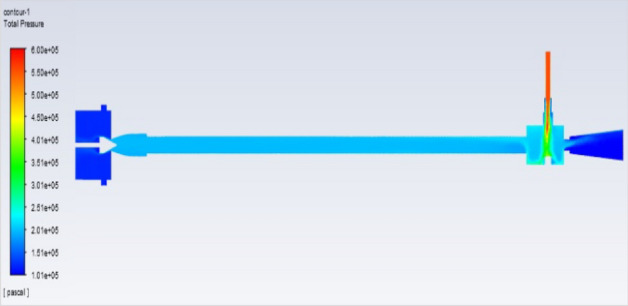
Pressure contours at 10 bar.

**Figure 56:**
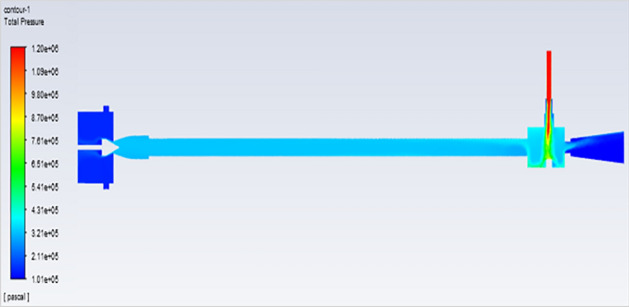
Pressure contours at 12 bar.

Figure 54 represents to vortex tube with inlet pressure as a maximum pressure of 8 bars (8,000,000 Pa). At the outlet the vortex tube has a minimum pressure of 101,325 Pa. Figure 55 represents to vortex tube with inlet pressure as a maximum pressure of 10 bars (10,000,000 Pa). At the outlet, the vortex tube has a minimum pressure of 101,325 Pa. Figure 56 represents to vortex tube with inlet pressure as maximum pressure of 12 bars (12,000,000 Pa). At the outlet, the vortex tube has a minimum pressure of 101,325 Pa.

### Vortex tube CAD details and the mesh elements

**Figure 57:**
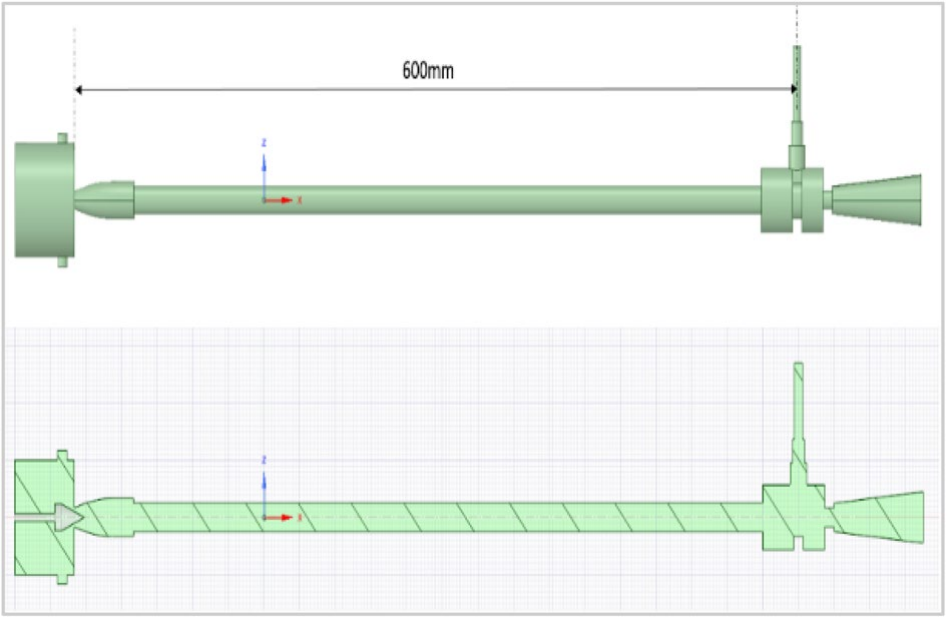
Vortex tube CAD details.

**Figure 58:**
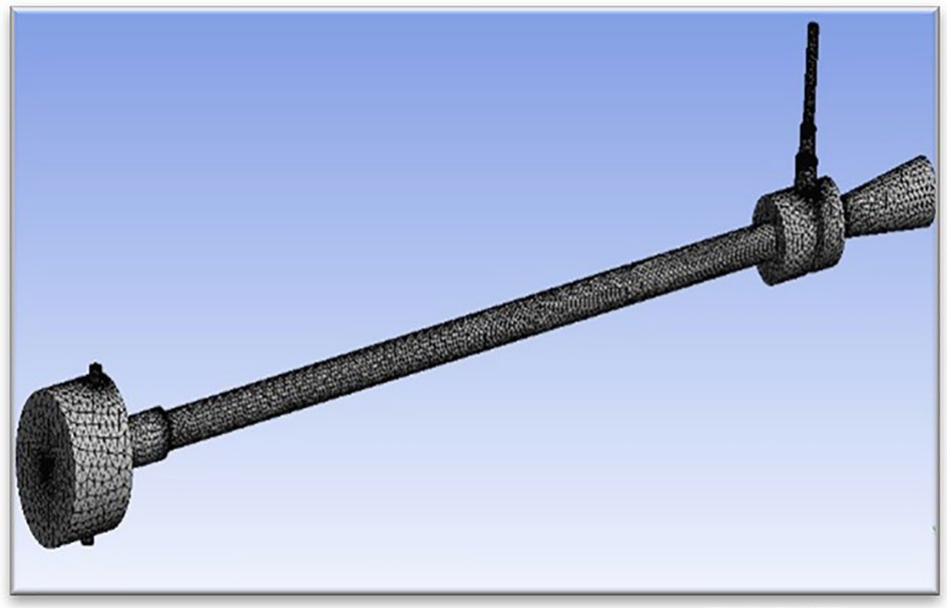
Ansys vortex tube model (mesh elements 3,17,553).

**Figure 59:**
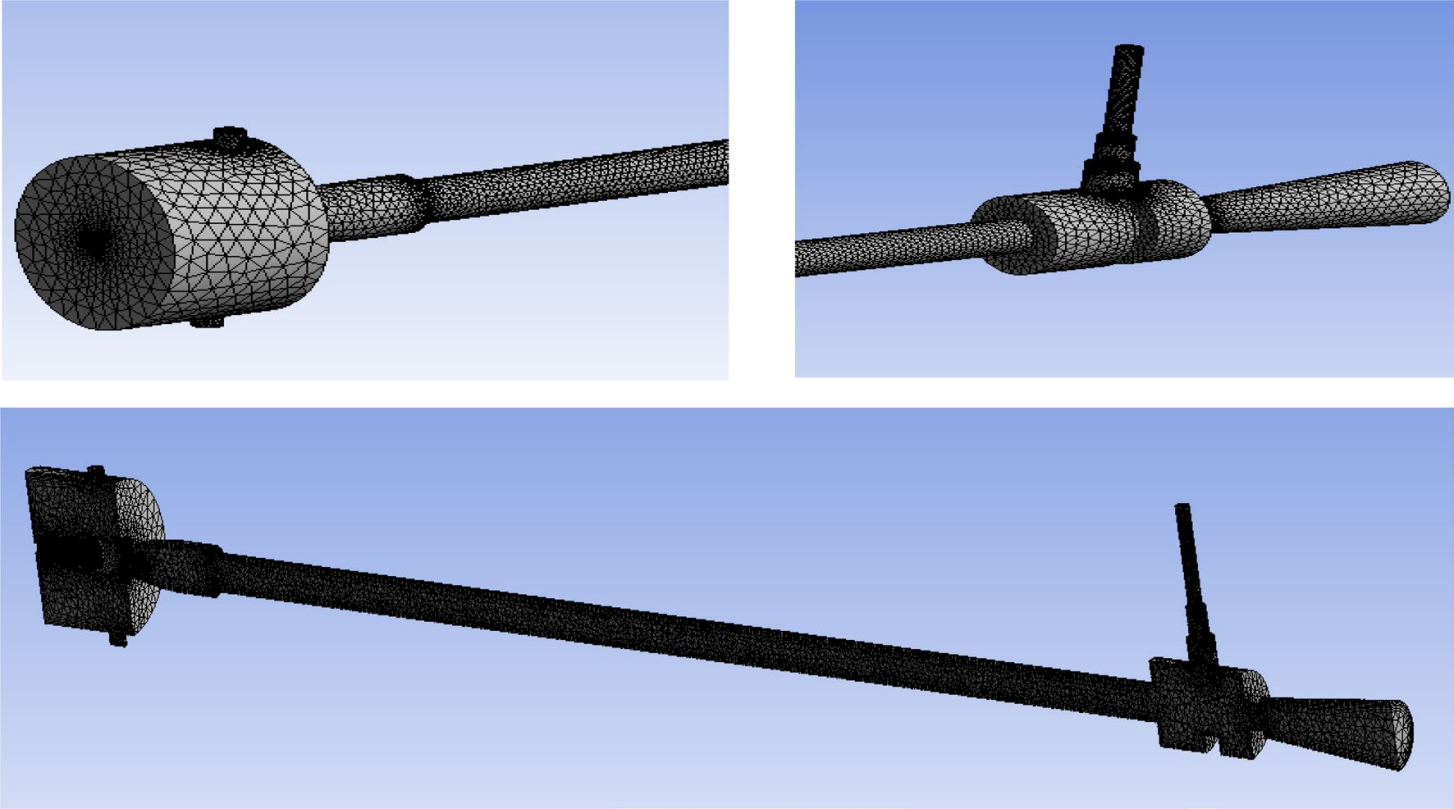
Schematic of vortex tube with grids of three-dimensional and cut section VT model.

Figure 57 explains about CAD details of the vortex tube with an L/D ratio of 40 for all eight types of material used in Vortex tube fabrication. (the length = 600 mm and the inner diameter of the hot tube was 15 mm was taken constantly) The pressure through the inlet varies from 2 to 12 bars. Figure 58 explained the computational model in Ansys with mesh elements 3,17,553. The computational model uses tetrahedral meshing. In Fig. 59 the computational domain and model solver conditions mentioned with a schematic of vortex tube with grids of three-dimensional and cut section of VT model is presented.

## Computational domain and model solver conditions

### Mesh independent study

**Figure 60:**
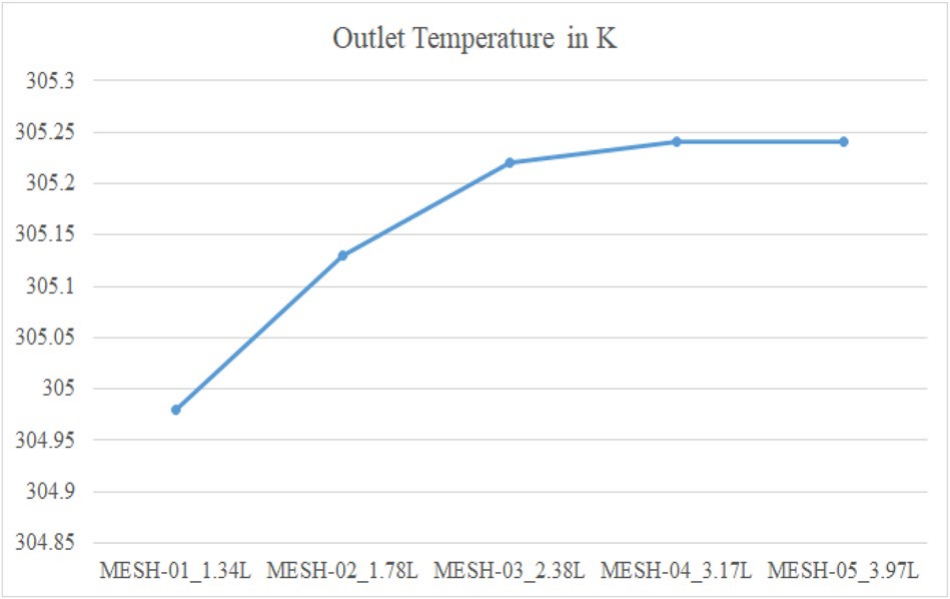
Mesh independent study.

Figure 60 shows the mesh independence study. The tetrahedral meshed in this study. To assure that the obtained results are independent of the mesh density, a mesh independent study was also carried out, Mesh 04 was selected based on the observation of hot outlet static temperature as shown (305.24 K). After 317,553 elements there is no significant changes in results were observed even after an increase in mesh size. Around 25% increase in mesh size was considered for each iteration of the mesh independent study shown in Table 1. Therefore, a mesh with 317,553 elements is used to reduce the computational time.

**Table 1:** The mesh independent study.

Mesh independent study	Mesh elements	Outlet temperature in K
MESH-01_1.34L	133,967	304.98
MESH-02_1.78L	178,623	305.13
MESH-03_2.38L	238,164	305.22
MESH-04_3.17L	317,553	305.24
MESH-05_3.97L	396,941	305.24

### Boundary condition

**Figure 61:**
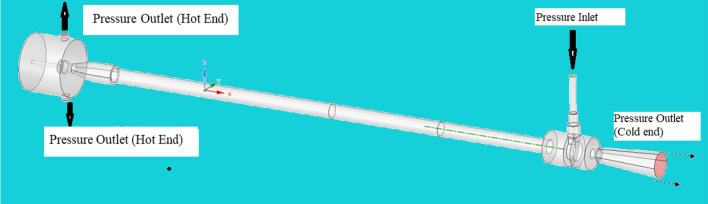
Boundary condition of vortex tube.

According to the CFD model, the inlet boundary can be as follows: the inlet acts as a pressure inlet; total pressure 200,000 Pa, air temperature 303 K. At the outlet, the cold end pressure is set to atmospheric pressure, and the hot end pressure is set to 101,325 Pa as shown in Fig. 61. In fluent, boundary conditions can be created in two steps, the first is to determine the boundary conditions in the meshing section, which is the type of material (classified as fluid, solid media, porous) in each sub-model, position. Inlet/outlet of the fluid model and type, wall boundary condition, etc. The important steps in the simulations are depending on the experiment results. The first one is to preset the boundary condition in the meshing section, such as the material type (divided as fluid, solid, porous media) in every sub-model, the position of the inlet/outlet of the fluid model and its type, the boundary condition of the wall, etc. Key steps in the simulation depend on the test results. The first one is to preset the boundary condition in the meshing stage, such as the type of material (classified as fluid, solid, porous media) for all sub-models, the inlet/outlet of the fluid model, and its type, the wall boundary condition, etc.

### Setup details

1. The gas in the vortex tube is assumed to be an ideal gas.
2. Set the reference pressure to 101,325 Pa, gravity is not considered.
3. The inlet of the vortex tube is set as pressure inlet, and pressure P_in_ = 4 bar, inlet pressure T_i_ = 303 K.
4. The cold source of the vortex tube is set to Pc = 0.1 MPa.
5. Hot source of the vortex tube is set to Ph = 0.1 MPa.
6. The outer wall of the vortex tube is mounted on a vertical wall.

#### Initialization from the inlet of the vortex tube

**Figure 62:**
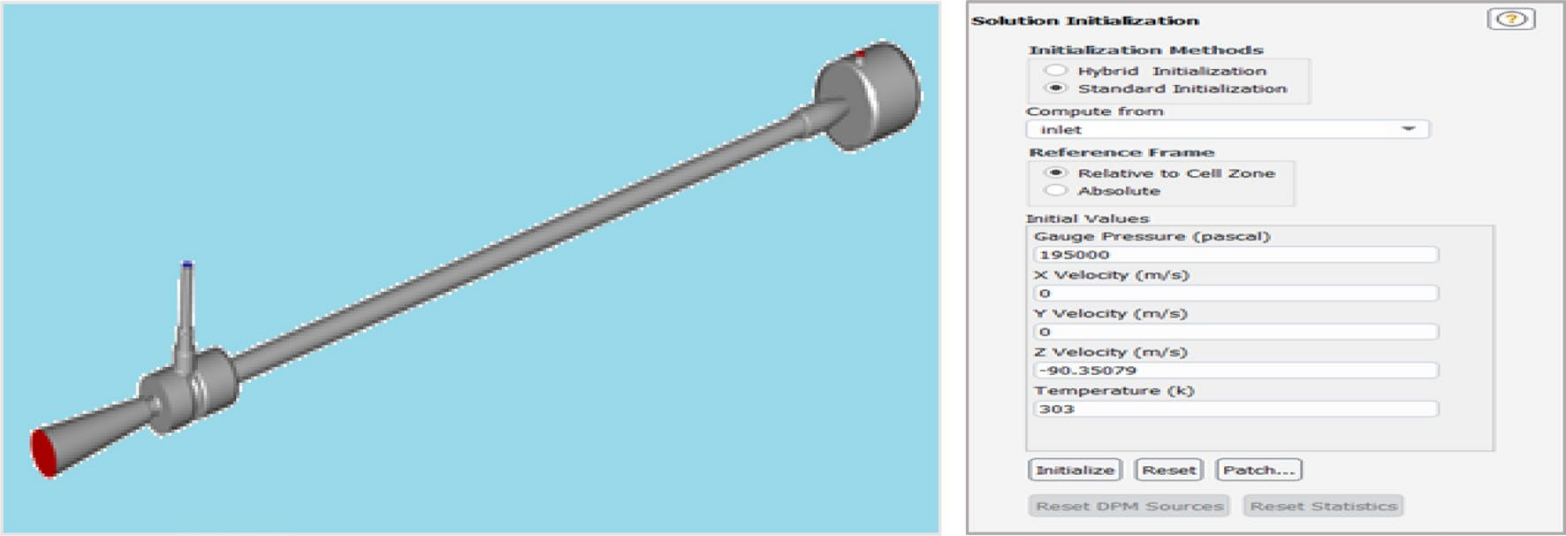
Initialization from the inlet of the vortex tube.

Initialization provides a quick evaluation of the stream field with a set of methods. All other variables, such as temperature, turbulence-type fraction, volume-type fraction, volume fraction, etc., will be automatically interpolated based on the average value of the domain or a specific interpolation method shown in Fig. 62.

**Figure 63:**
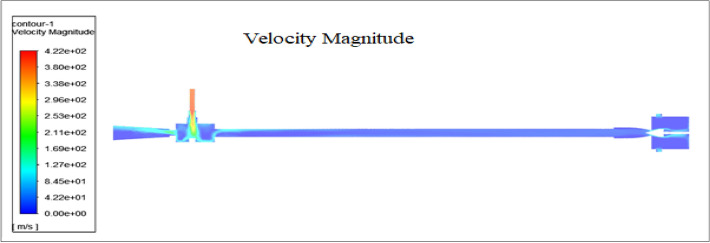
Velocity magnitude of the vortex tube.

Standard initialization allows directly defining all variables as initial values. Fluent is configured that can easily set the initial x-velocity, y-velocity, z-velocity, temperature, pressure, etc., a constant field throughout the domain. Some work is required to specify the continuous fields, but you can consider specifying the normal spatial distribution of all variables (Fig. 63).

### Mach number (M)

Mach number is an important ratio term that we use when moving through the air. It is a ratio of the speed of flow behind certain limits of the speed of sound. In other words, it is the ratio of the speed of sound to the speed of sound in the surrounding medium.

Mach number = Speed of flow/speed of sound in air

$$\mathrm{M }=\mathrm{ v}/\mathrm{c}$$

Let:

M = Mach number.

v = speed of the object.

c = Speed of sound in air (342 m s^−1^).

Mach number at inlet: 1.13.

Mach number at cold outlet: 0.18.

Mach number at hot outlet: 0.23.

### Velocity magnitude

**Figure 64:**
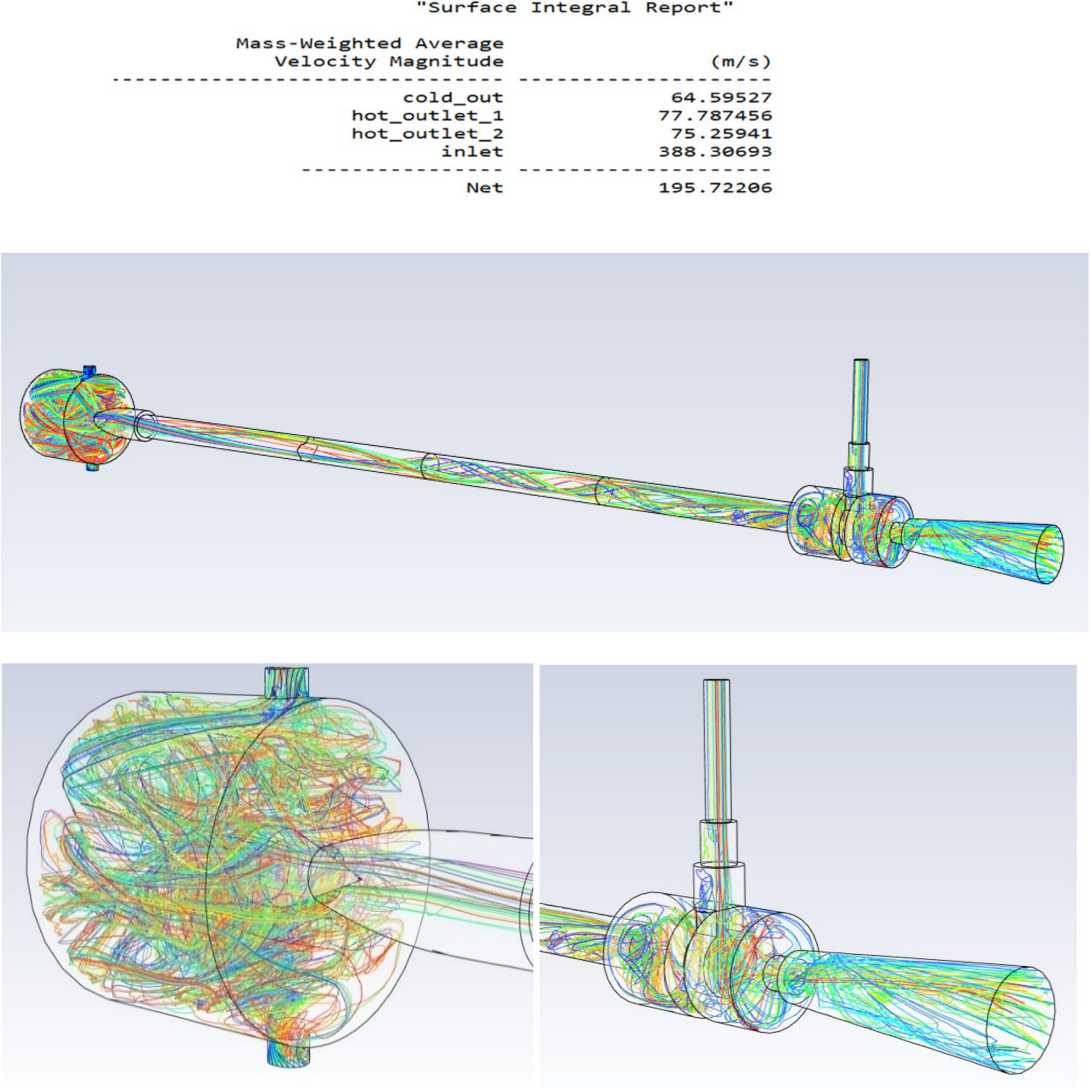
Streamlines of the vortex tube.

The above-mentioned Fig. 64 is about the streamlines of the vortex tube at 12 bar pressure at L/D ratio of 40.

### Validation

**Figure 65:**
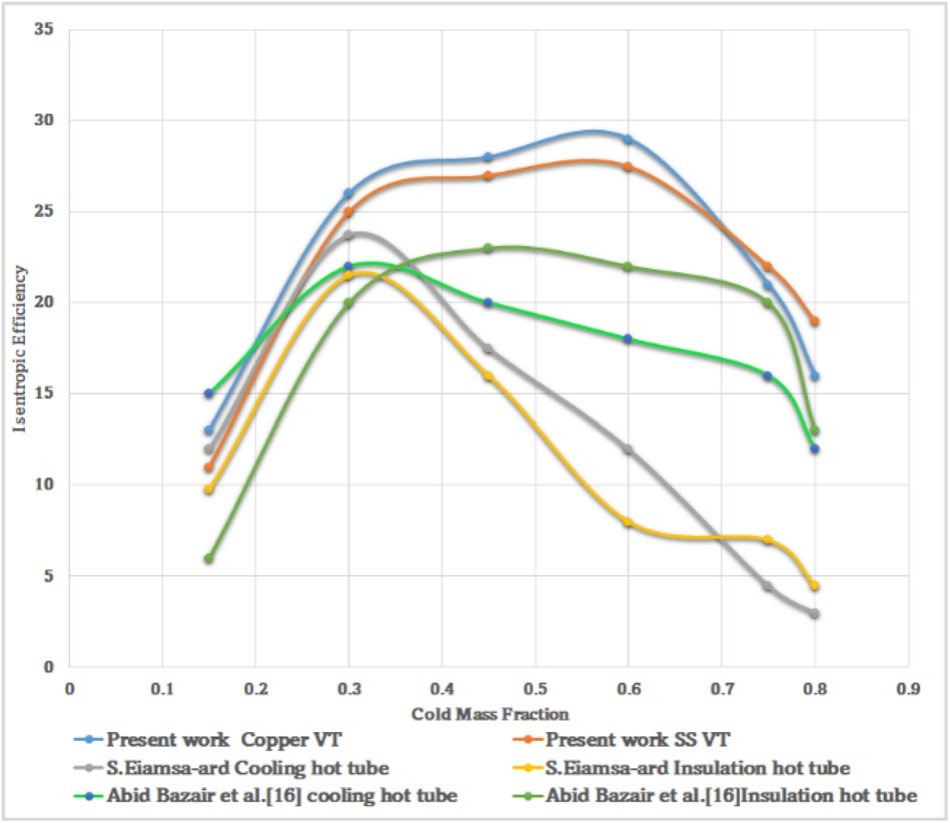
CMF vs isentropic efficiency.

To evaluate the research results, all data has been validated and compared with the results of Eiamsa-ard et al.^40^ and Bazair et al.^16^ with insulation, and cooling hot tubes. The Isentropic efficiency versus cold mass fraction has been plotted in Fig. 65. As shown, the results at different cold mass fractions were presented both by experimental data and by numerical results. The results show the variation pattern of isentropic efficiency versus cold fraction. In the present work, the researcher observed improved results compared to previous work achieving the highest isentropic efficiency for cold fraction, between 0.1 and 0.8.

**Figure 66:**
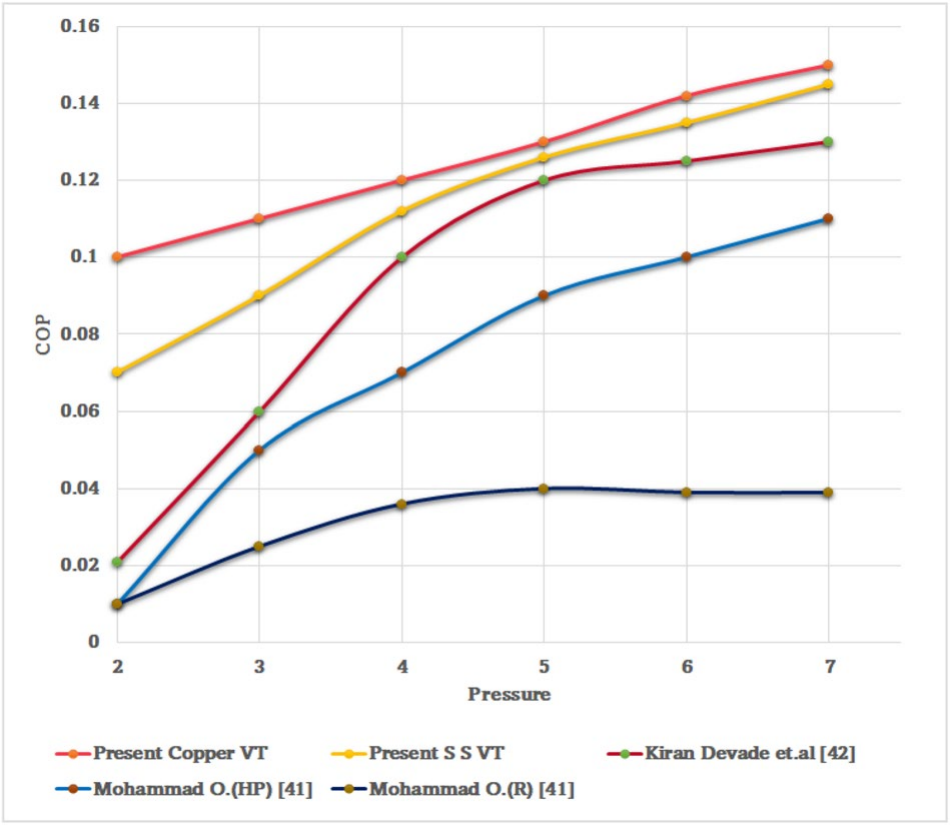
Pressure vs COP.

From Fig. 66 it is seen clearly that the COP increases almost singularly with the pressure. It is clear that the COP is directly proportional to inlet pressure. It was also observed that in Fig. 66 the CFD simulation and experimental results of Devade^42^ and Mohammad^41^. For all test runs, the COP was significantly improved with an increase of inlet pressure ranging from 2 to 7 bars. The present CFD simulations agree well, and confirms with these experimental results.

## Results and discussion

The geometrical specification like length to diameter ratio (L/D = 40), nozzle diameter (D_n=_ 10 mm), orifice diameter (d_c_ = 8 mm), and different materials like metals and non-metals were analyzed on maximum temperature difference (∆T = T_h_ − T_c_), isentropic efficiency (η_Is_), adiabatic efficiency(η_ad_) and COP of Vortex tubes. All these results are analyzed with pressure variations; a cold mass fraction, isentropic efficiency (η_Is_), and adiabatic efficiency (η_ab_) are utilized for the analysis in the range of pressure specified in the results. The results were presented along with the associated literature, and results obtained during the simulation and experimental analysis were presented in Figs. 3, 4, 5, 6, 7, 8, 9, 10, 11, 12, 13, 14, 15, 16, 17, 18, 19, 20, 21, 22, 23, 24, 25, 26, 27, 28, 29, 30, 31, 32, 33, 34, 35, 36, 37, 38, 39, 40, 41, 42, 43, 44, 45, 46, 47, 48, 49 and 50 clearly about inlet temperature, hot end temperature, and cold end temperature with inlet pressures from 2 to 12 bars with the step of 2 bars.

### Outcome of pressure vs adiabatic efficiency of materials

**Figure 67:**
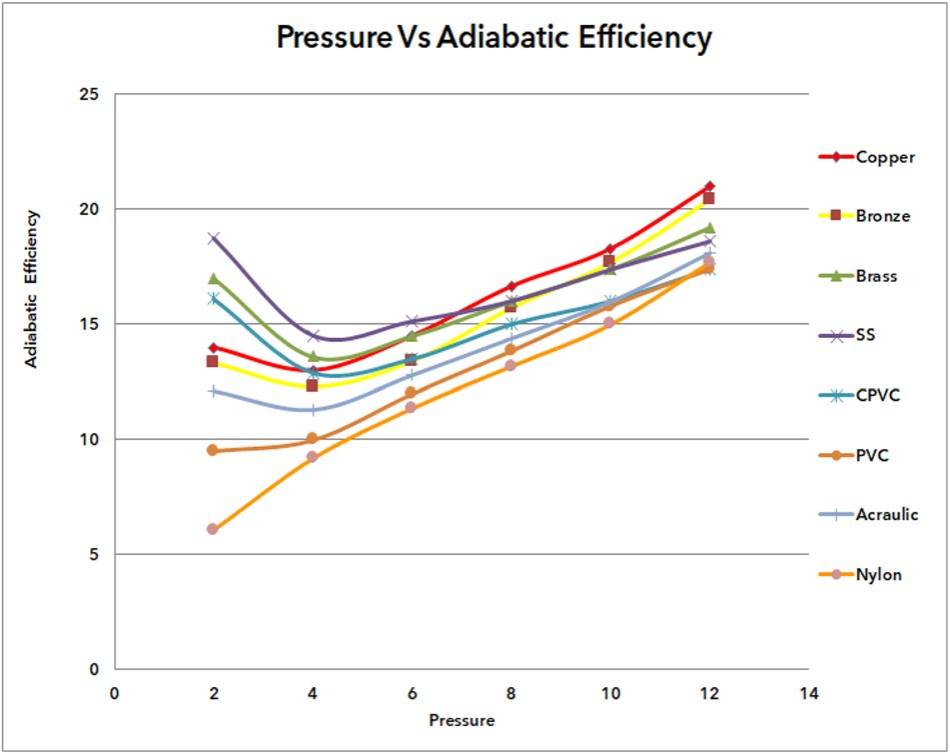
Pressure vs adiabatic efficiency.

Figure 67 shows the adiabatic efficiency of various materials, from copper to nylon, with inlet pressures ranging from 2 to 12 bars. It was observed that adiabatic efficiency was improved gradually from nylon to copper. The least adiabatic efficiency was obtained as 6.09% at 2 bar pressure with nylon material; in addition, the maximum efficiency of copper was 21% at 12 bar pressure. It was noticed from the results from copper to nylon the adiabatic efficiency was declining. The materials in descending order of adiabatic efficiency from copper, bronze, brass, stainless steel, CPVC, PVC, acrylic, and nylon.

### Outcome of pressure vs isentropic efficiency of materials

**Figure 68:**
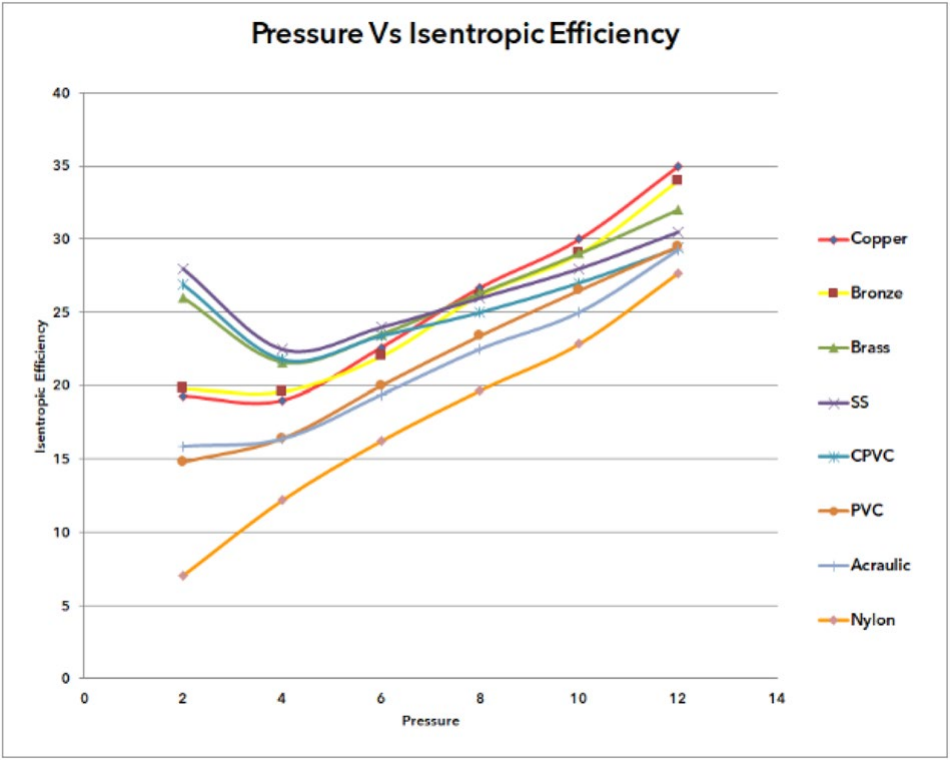
Pressure vs isentropic efficiency.

Figure 68 shows the pressure versus isentropic efficiency of various materials with inlet pressure at the entrance of the vortex tube varies from 2 to 12 bars for a range of materials in the results from copper to nylon. It was observed that the isentropic efficiency has gradually improved from nylon to copper material. The lowest isentropic efficiency of 7.06% was noticed for nylon at 2 bar pressure; In addition, at 12 bar pressure, the maximum efficiency was observed for copper material as 35%.

### Outcome of pressure vs maximum temperature difference of materials

**Figure 69:**
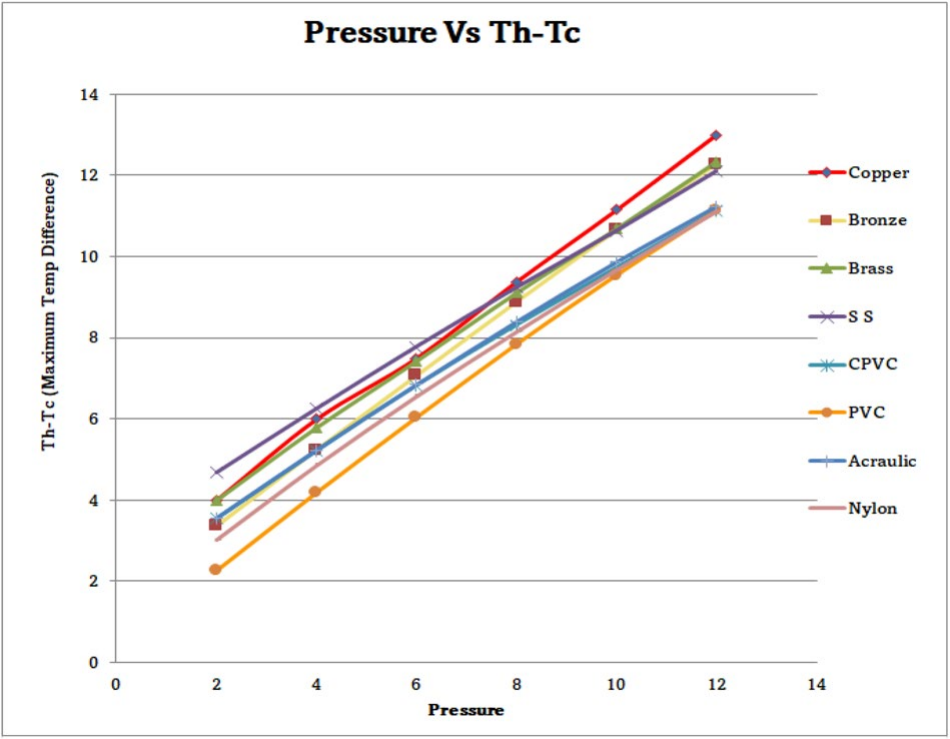
Pressure vs maxi temp difference.

Figure 69 represents the outcome of pressure with a consequence of maximum temperature difference (∆T) with different materials. It was observed that the ∆T was 13°C maximum at 12 bar pressure for the copper vortex tube. Similarly, the least difference was noticed as 2.26 °C at 2 bar pressure with PVC material VT. By the results, it was noticed that while pressure increased gradually from 2 to 12 bars, the temp difference also increased gradually for all materials.

### Outcome of pressure Vs COP of materials

**Figure 70:**
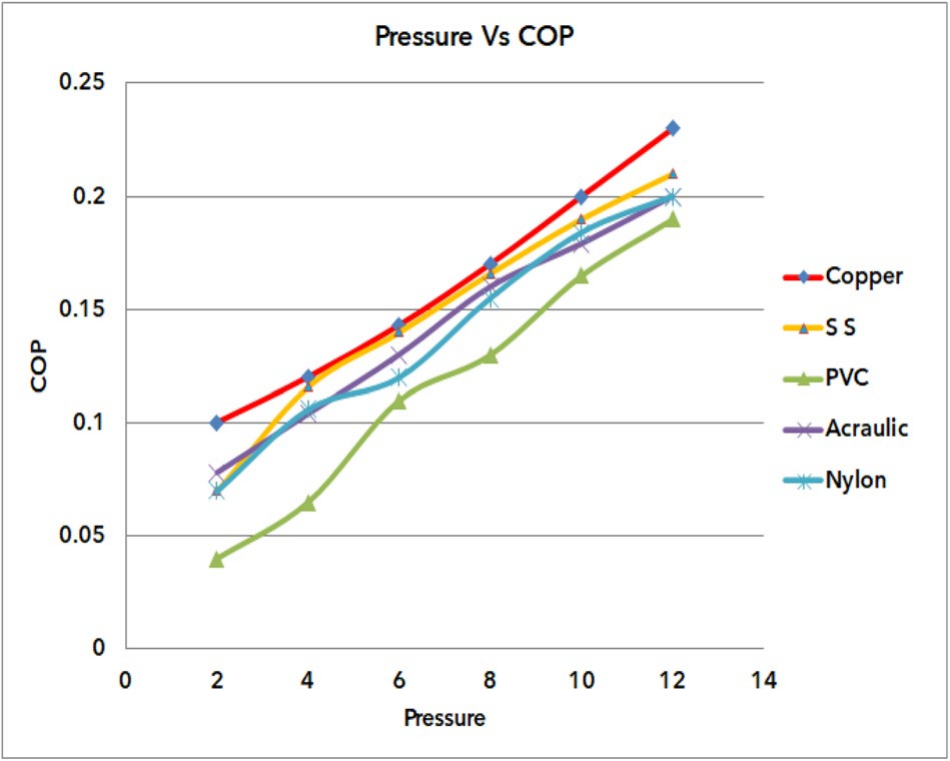
Pressure vs COP.

Figure 70 represents the outcome of pressure versus COP of different materials of vortex tubes. The simulation was performed at the entrance with an inlet pressure of 2–12 bars in steps of 2 bars. The maximum COP of 0.23 was obtained at a pressure of 12 bar with a copper vortex tube. At the same time, a minimum COP of 0.07 was noticed for the nylon vortex tube. While the pressure at the entrance of the vortex tube increases, the temperature difference between the cold terminal and hot terminal increases. As the inlet pressure increases, the transfer of kinetic energy is found to increase the energy separation causing raise in the COP of the VORTEX tube. For the easy identification purpose only five materials were considered (copper. SS, PVC, acrylic, nylon, etc.,) in the graph.

### Outcome of CMF vs adiabatic efficiency of materials

**Figure 71:**
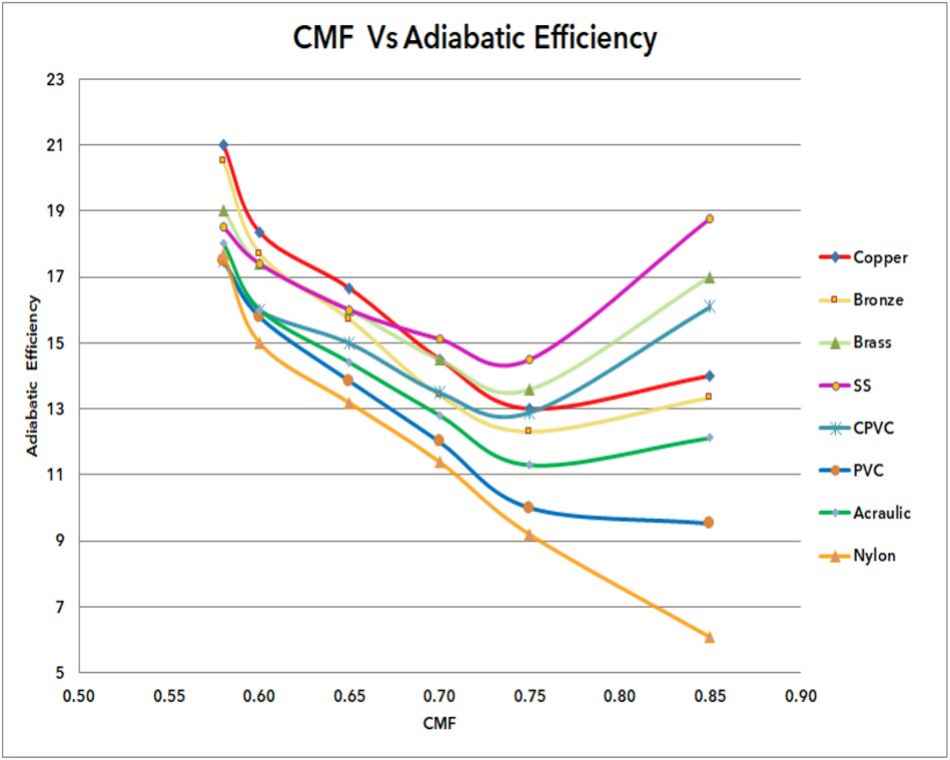
CMF vs adiabatic efficiency.

Figure 71 shows the outcome of CMF Vs adiabatic efficiency of materials. From the graph, it was observed that the adiabatic efficiency of various materials under cold mass fraction ranges from 0.58 to 0.85. It can be observed that the adiabatic efficiency has gradually improved from nylon to copper. The lowest adiabatic efficiency was 6% for nylon was observed at 0.85 CMF; similarly, the maximum efficiency was found to be 21% at 0.58 CMF. While at maximum CMF the efficiency has decreased, similarly at minimum CMF the efficiency was found to be maximum.

### Outcome of CMF vs isentropic efficiency of materials

**Figure 72:**
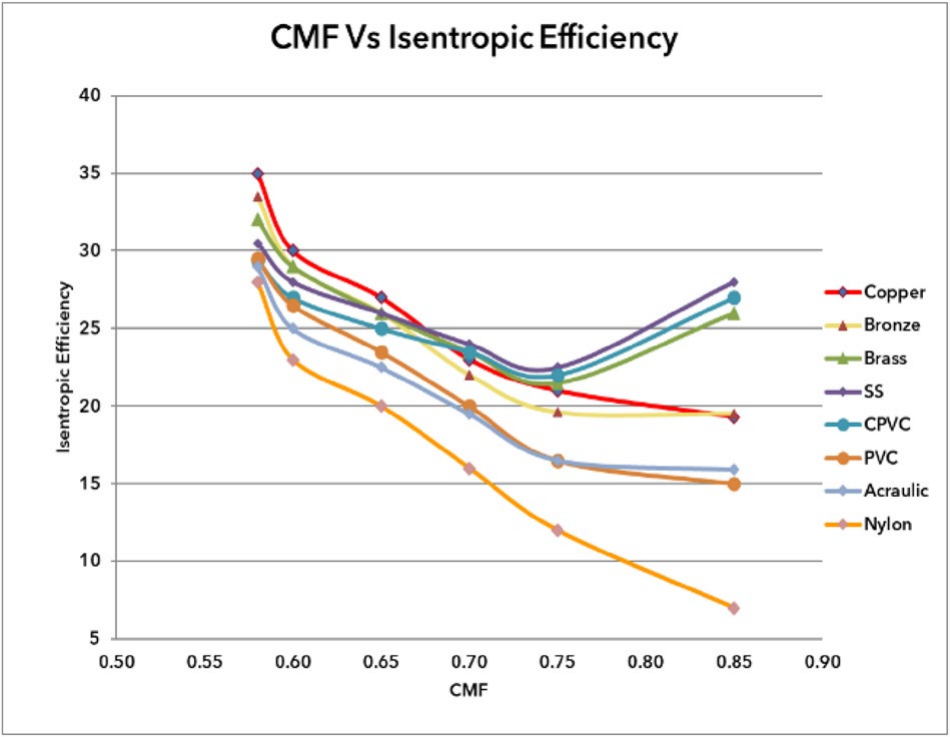
CMF vs isentropic efficiency.

Figure 72 shows the isentropic efficiency of various materials under cold mass fractions from copper to nylon. It is observed from the fig that the isentropic efficiency has gradually improved from nylon to copper material. The lowest isentropic efficiency was noticed as 7% with nylon at 0.85 of CMF; similarly, the maximum efficiency for Copper was found to be 35% at 0.58 of CMF.

## Properties of materials

The below mentioned Table 2 explains the material properties of eight materials that are used in CFD simulation to evaluate and find the optimum material to fabricate the vortex tube with density, melting point, and modulus of elasticity, thermal conductivity, etc.

**Table 2:** Materials proprties of different materials used in the present research work.

Sl. no	Material	Density ( kg m^−3^)	Melting point (°C)	Modulus of elasticity (GPa)	Thermal conductivity ( W m^−1^ K^−1^)
1	Copper	8940	1084.62	117	386
2	Bronze	8700	950	120	26
3	Brass	844 0	916	103.4	116
4	SS	7500	1450	193	15
5	CPVC	1450	150	2.52–3.10	0.139
6	PVC	1467	260	0.003–4.83	0.33
7	Acrylic	1.19	160	3.2	0.2
8	Nylon	1.15	220	2.7	0.25

## Effect of the pressure on temperature difference, isentropic and adiabatic efficiencies, and COP

**Figure 73:**
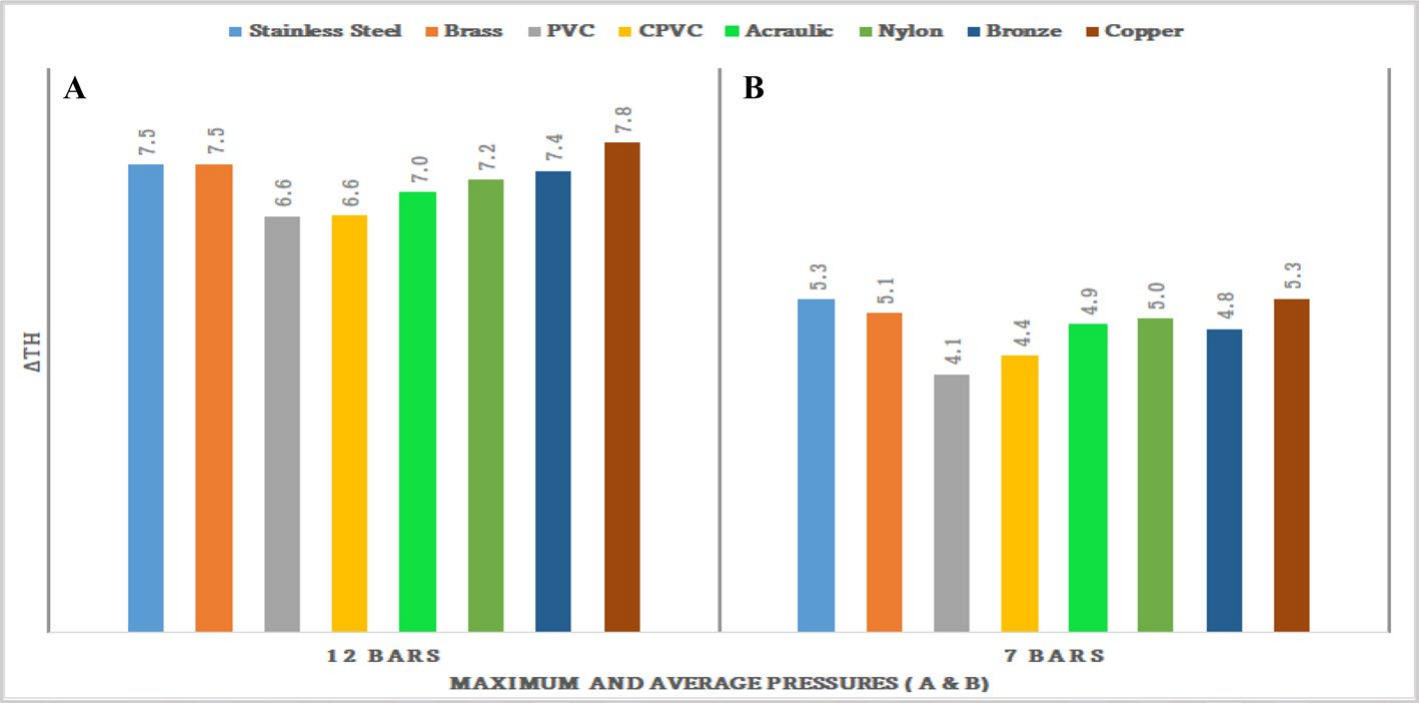
Hot end temperature difference versus maximum and average pressures.

**Figure 74:**
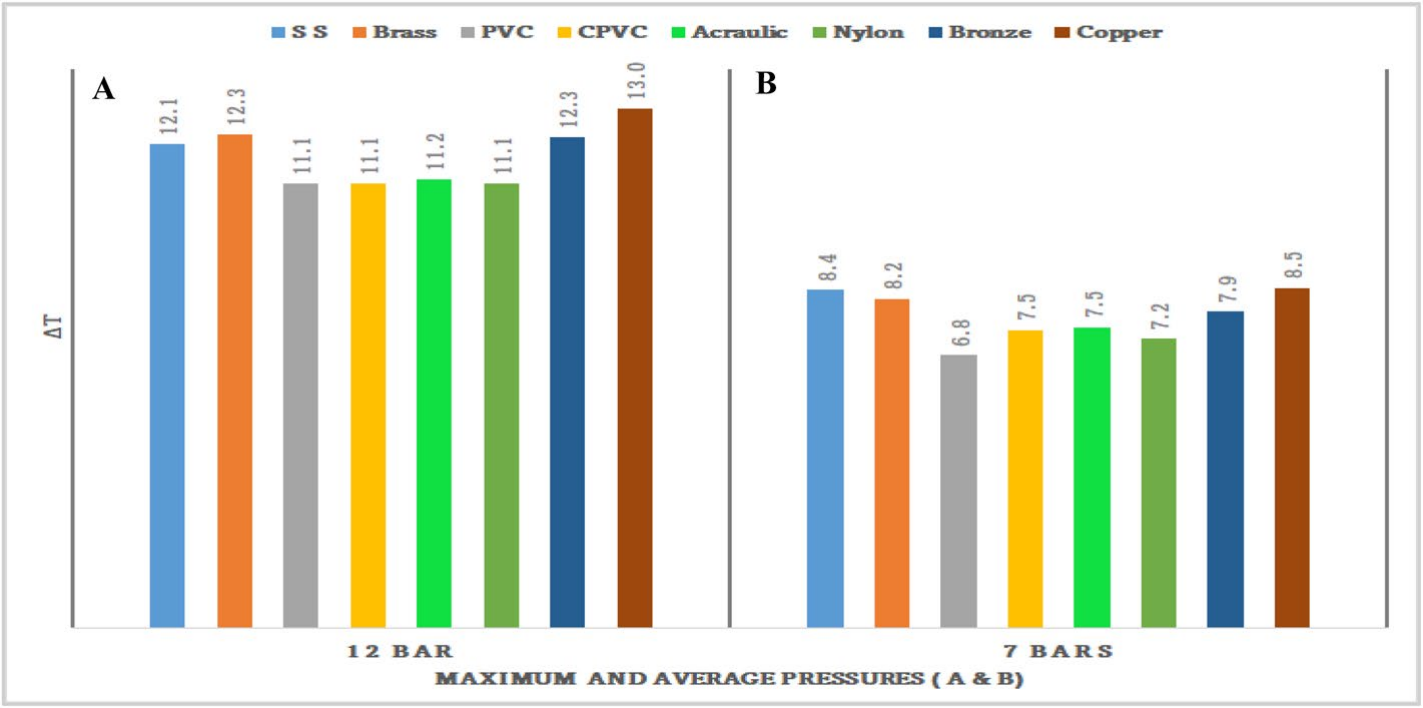
Maximum and average pressures versus maximum temperature (∆T) difference.

**Figure 75:**
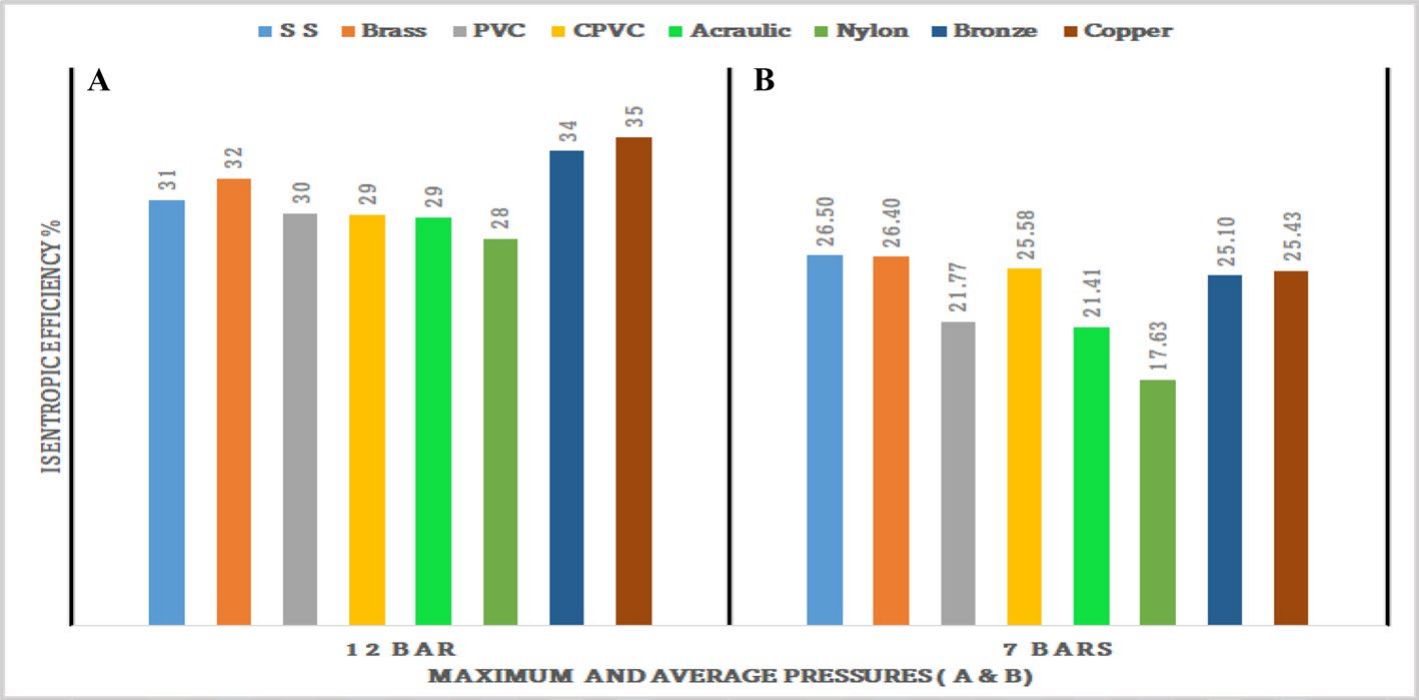
Maximum and average pressure versus isentropic efficiencies (η_Isen_).

**Figure 76:**
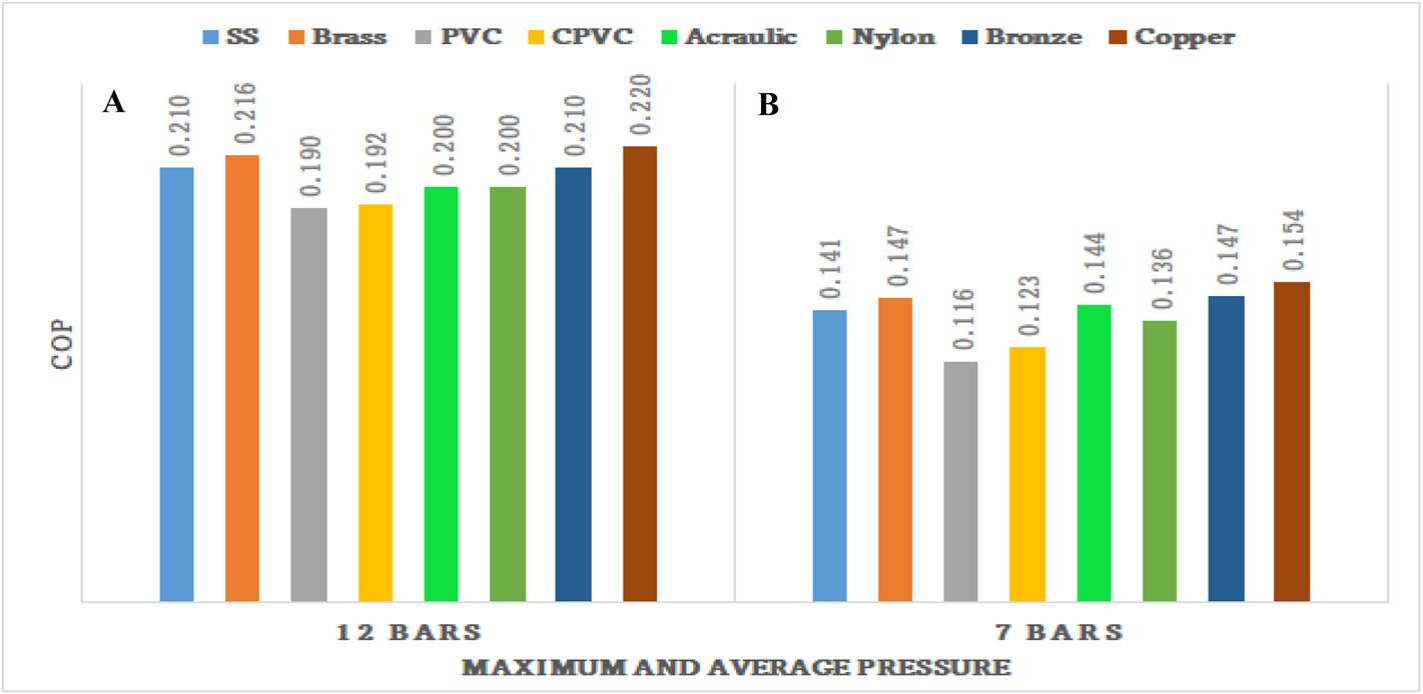
Maximum and average pressures versus COP.

**Figure 77:**
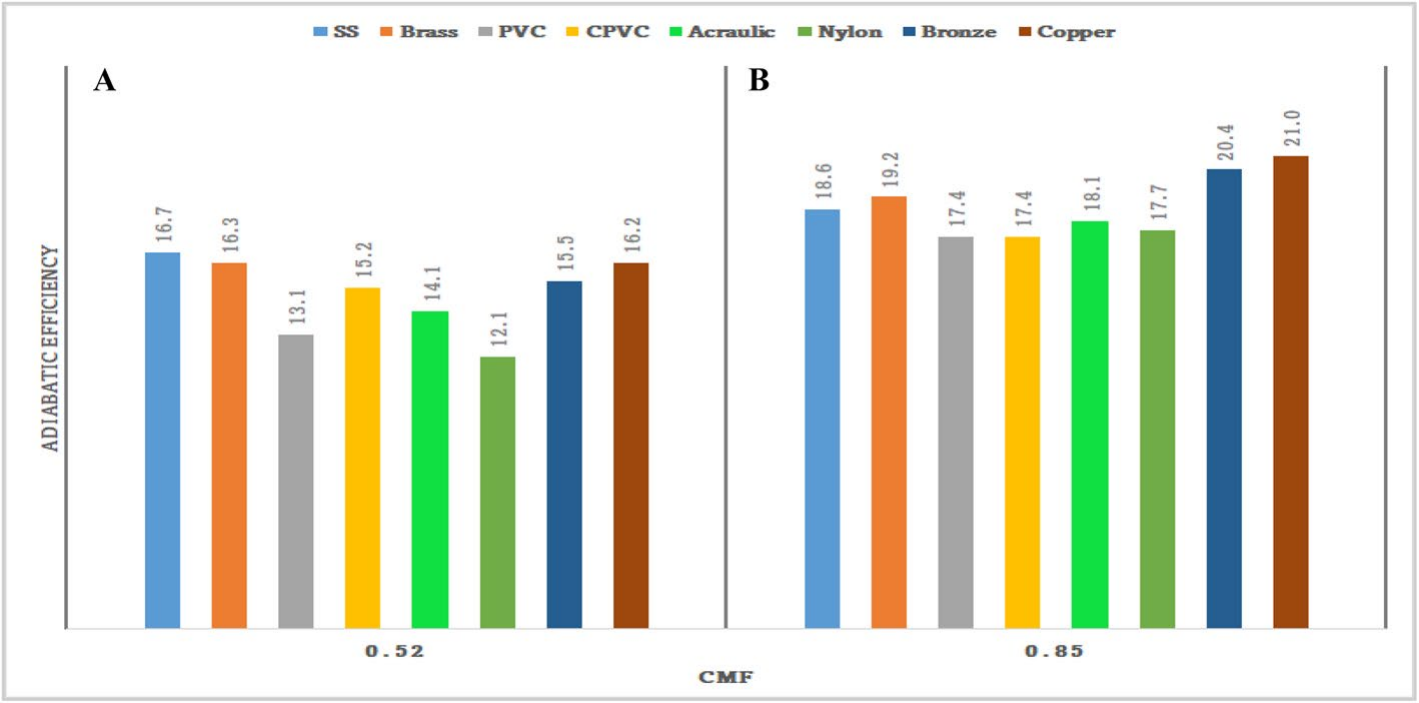
Average and maximum CMF versus adiabatic efficiencies (η_adia_).

**Figure 78:**
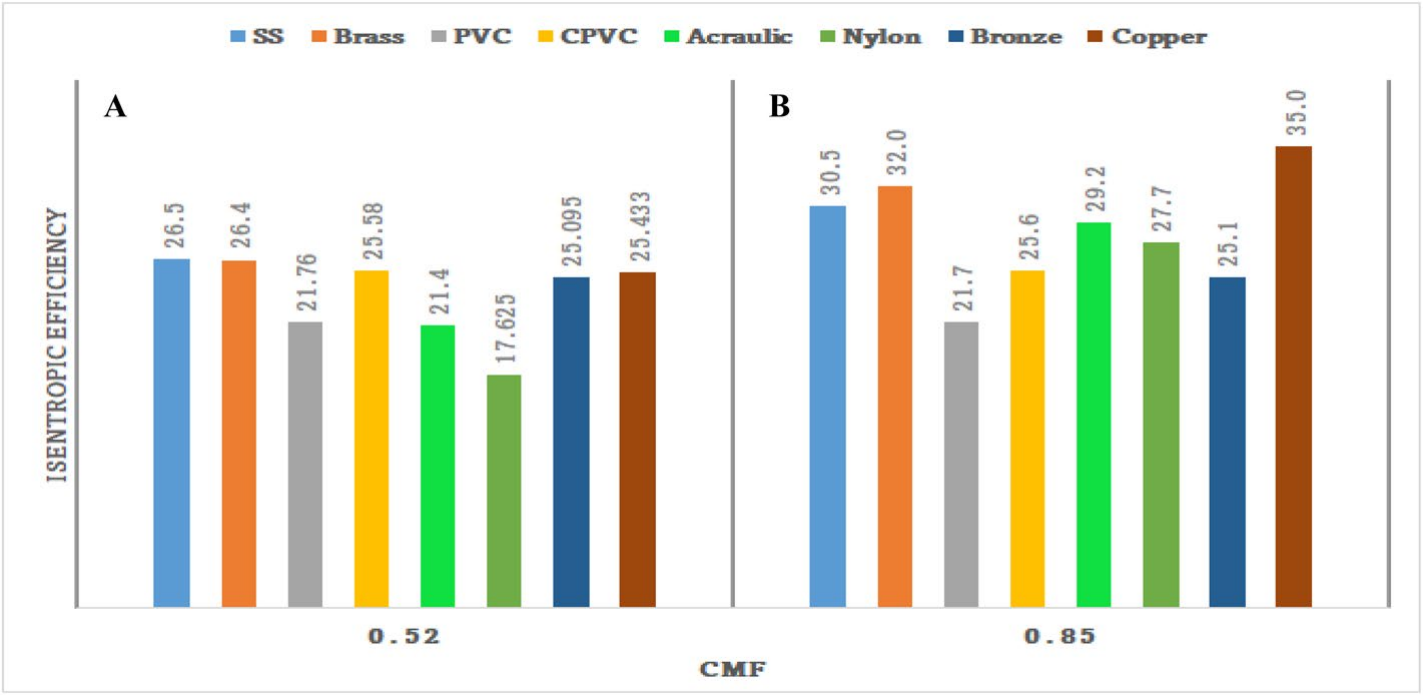
Average and maximum CMF versus isentropic efficiencies (η_Isen_).

Figures 71, 72, 73, 74, 75 and 76 represents the effect of the pressure on hot end temperature difference, Maximum temperature difference, Isentropic efficiency, Adiabatic Efficiency, and COP for different materials simulation analyses of Vortex tube are explained. In this connection, the Maximum and Mean (Average) pressures are taken into the consideration. From Figs. 77 and 78 the effect of the CMF verses, Adiabatic and Isentropic efficiencies are presented respectively.

### Effect of the pressure on hot end temperature difference

Figure 73 shows the effect of the pressure on the hot end temperature. Figure 73A is about maximum pressure versus hot end temperature difference. From the fig, it is clear that the highest temperature of 7.8 °C is recorded by the copper-made Vortex Tube. Similarly for stainless steel and brass made vortex tubes the hot end temperature of 7.5 °C was attained. The least temperature difference was noticed as 6.6 °C for CPVC-made VT. Similarly, Fig. 73B represents the average pressure (7 bars of mean pressure) versus the hot end temperature. The highest temperature of 5.3 °C is recorded for the copper and SS made vortex tubes. The least temperature difference was attained for PVC-made vortex tube as 4.1 °C.

### Effect of the average and maximum pressure on maximum temperature difference

Figure 74 represents the effect of the pressure on maximum temperature (∆T). Figure 74A is about Maximum pressure versus hot end temperature difference. The highest temperature of 13 °C is recorded by the copper-made vortex tube. Stainless steel and brass made vortex tubes attained the hot end temperature of  12.1 °C and 12.3 °C respectively.

Similarly, Fig. 74B represents the average pressure (7 bars of mean pressure) versus the hot end temperature. The highest temperature of 8.5 °C and 8.4 °C is recorded by the copper and SS-made vortex tubes respectively. The least temperature difference is recorded by PVC-made vortex tube at 6.8 °C.

### Effect of the average and maximum pressure on isentropic efficiency

Figure 75A represents maximum pressure versus isentropic efficiency. At the maximum pressure, the highest isentropic efficiency of 35% is recorded by the copper-made vortex tube. Bronze, stainless steel, and brass made vortex tubes attained the hot end temperature of 34%, 31%, and 32% respectively.

Similarly, Fig. 75B represents the average Isentropic efficiency of 26.5%, 26.4%, and 25.43% as recorded by the SS, brass, and copper vortex tubes respectively. The least isentropic efficiency is recorded by nylon-made vortex tube at 17.63%

### Effect of the average and maximum pressure on COP

Figure 76A represents Maximum pressure versus COP. At the maximum pressure, the highest COP of 0.22 is recorded by the copper-made vortex tube. bronze, stainless steel, and brass made vortex tubes attained the COP at O.216, 0.21, and 0.21 respectively.

Similarly, Fig. 76B represents the average COP of 0.154 and 0.147, which are recorded by the copper, brass, and bronze vortex tubes respectively. The minimum COP is recorded by CPVC-made vortex tube at 0.123. The least COP is recorded by nylon-made vortex tube at 0.116.

### Effect of the CMF versus adiabatic efficiency

Figure 77A represents average CMF versus adiabatic efficiency. At the average CMF, the highest adiabatic efficiency of 16.7% is recorded by the SS-made vortex tube. Brass and copper-made vortex tubes attained COP of 16.3%, and 16.2% respectively.

Figure 77B represents the maximum CMF versus adiabatic efficiency. At the maximum CMF, the highest adiabatic efficiency of 21% is recorded by the copper-made vortex tube. brass and copper-made vortex tubes attained the COP of 19.2%, and 20.4%, respectively. The least adiabatic efficiency is recorded by CPVC and nylon-made vortex tube as 17.4%.

### Effect of the CMF versus Isentropic efficiency

Figure 78A represents average CMF versus isentropic efficiency. At the CMF, the average adiabatic efficiency of 26.5% is recorded by the SS-made vortex tube. Brass and copper-made vortex tubes attained adiabatic efficiency of 26.4%, and 25.43% respectively. Figure 78B represents maximum CMF versus isentropic efficiency. At the maximum CMF, the highest adiabatic efficiency of 35% is recorded by the copper-made vortex tube. SS, brass-made vortex tubes attained the adiabatic efficiency of 30.5%, and 32% respectively. The minimum value is recorded by PVC-made VT as 21.7%.

After observation of all the above figures for different materials used in the fabrication of the vortex tubes the following observations were drawn:

- From Figs. 73, 74, 75 and 76 it was evident that the pressure is the driving force for all factors influencing the energy separation process inside the Vortex tube^42^. The results are shown that the maximum temperature difference and a higher COP are attained with the increase in inlet pressure.
- From Figs. 73 and 74 it was evident that as the temperature difference increases, the energy separation decreases with the increase in inlet pressure^37^.
- From Fig. 75 it was evident that^44^ the cold air temperature difference increases by increasing the inlet pressure, meanwhile, there is an optimum efficiency at specific inlet pressure.
- From Figs. 77 and 78, it was evident that temperature reduction and Isentropic efficiency (cooling efficiency) significantly increase with the increase of cold mass fraction^41^.
- It is important to note that for all feasible operations of the Vortex tube, the choice of durable material for the manufacture of the tube is also quite important^44^.
- All investigators concluded that using materials with more smooth surfaces and lower thermal conductivities (according to Table 2. SS thermal conductivity is 15 W/m. K attains the best performance from the simulation) and using the tube with insulation to reduce energy loss to surroundings results in better temperature separation and performance^38,39^.

## Conclusions

In this work, the executive parameters were analyzed for the Vortex tube by using CFD simulation for a range of materials. By the results, the optimal material of the Vortex tube considering the values of isentropic efficiency, adiabatic efficiency, COP, and maximum temperature difference was determined.

1. The maximum COP was recorded as 0.22 by copper vortex tube with an intake pressure of 12 bars. Similarly at that pressure, the minimum COP of 0.19 was recorded for PVC material.
2. It was observed that the maximum isentropic efficiency obtained was 35% at 12 bar pressure of inlet for Copper Vortex tube and a minimum isentropic efficiency value of 27.7% was recorded for the Nylon material. Similarly, at 2 bar intake pressure, it was observed least isentropic efficiency was recorded for nylon material at 2 bar.
3. The maximum temperature difference noticed was 13 °C at 12 bar pressure for copper material, similarly at the same pressure the least temperature difference was obtained as 11.11 °C for the nylon material VT.
4. The maximum adiabatic efficiency of 21% was obtained at 12 bar pressure for Copper Material, likely at the same pressure for both PVC and CPVC material the adiabatic efficiency of 17.5%was recorded.
5. The pressure is the most important in the role of increasing COP, maximum temperature differences, adiabatic efficiency, and isentropic efficiency. It is very clear that with the increase in inlet pressure the performance also improved.
6. The maximum adiabatic efficiency of 21% was obtained at 0.58 CMF for copper material, likely the minimum adiabatic efficiency was recorded as 6.09% at 0.85 CMF for nylon material.
7. The maximum isentropic efficiency of 35% was obtained at 0.58 CMF for copper material, similarly, the minimum adiabatic efficiency was recorded as 7% at 0.85 CMF for nylon material.
8. Finally, after evaluating all simulation values for eight materials VT performance which depend upon their physical properties, varies in the sequence from copper–bronze–brass–stainless steel–CPVC–PVC–acrylic–nylon material.
9. It is clear that the (non-copper material) SS-made VT with low thermal conductivity attains the best energy separation, efficiency and performance.

## Supplementary Information

Supplementary Information 1.

Supplementary Information 2.

## Data Availability

All data generated or analyzed during this study are included in this Manuscript article [along with its supplementary information files.

## List of symbols

CFD: Computational fluid dynamics
COP: Coefficient of performance
VT: Vortex tube
d: Diameter of hot tube (mm)
Cp: Specific heat at constant pressure (J kg^−^^1^ K^−1^)
L: Length of the hot tube (mm)
ṁ_c_: Cold mass flow rate (kg s^−1^)
P: Absolute total pressure (bar)
T: Temperature (K)
T_h_: Temperature of hot air
T_i_: Temperature of inlet air
T_c_: Cold end temperature
V: Velocity (ms^−1^)
d_c_: Diameter of cold orifice
D_n_: Nozzle diameter

## Greek symbols

γ: Specific heat ratio (Cp Cv^−1^)
η: Efficiency (dimensionless)
µ: Cold mass fraction
ρ: Density (kg m^−3^)

## Subscripts

i: Inlet
Is: Isentropic
a: Atmospheric
ad: Adiabatic
