# Supplementary material for: CFD simulation on optimum material to fabricate the counter flow of Ranque–Hilsch vortex tube
Source: Sci Rep. 2022 Sep 28;12:16226. doi: 10.1038/s41598-022-19779-0 (PMC9519905; doi:10.1038/s41598-022-19779-0)
Supplement: Supplementary file 1 — Supplementary Information 1. [file 41598_2022_19779_MOESM1_ESM.docx]

The given tables were represented for 8 different material Vortex tube simulation values were calculated from 2 -12 bars intake pressures at the inlet. The given below table were presented with

1. **Temperature difference 2. Isentropic Efficiency 3. Adiabatic Efficiency 4.COP**

**Table-S1: 2 bar pressure at inlet of the Vortex tube**

| L/D Ratio = 40 | |  |  |  |  |  |  |  |
| --- | --- | --- | --- | --- | --- | --- | --- | --- |
|  | **Stainless Steel** | **Brass** | **PVC** | **CPVC** | **Acraulic** | **Nylon** | **Bronze** | **Copper** |
| **Inlet Pressure in Bar** | **2** | | | | | | | |
| **Inlet Temperature in ^0^K** | **303** | **303** | **303** | **303** | **303** | **303** | **303** | **303** |
| **Cold Outlet** | **301.47** | **301.6** | **302.2** | **301.55** | **302.14** | **302.62** | **301.93** | **301.96** |
| **Hot Outlet 1** | **306.14** | **305.69** | **304.46** | **305.08** | **305.69** | **305.65** | **305.27** | **305.75** |
| **Hot Outlet 2** | **306.12** | **305.68** | **304.46** | **305.09** | **305.7** | **305.66** | **305.22** | **305.76** |
| **Inlet - Cold Out** | **1.53** | **1.4** | **0.8** | **1.45** | **0.86** | **0.38** | **1.07** | **1.04** |
| **Hot Out - Cold Out** | **4.67** | **4.08** | **2.26** | **3.54** | **3.55** | **3.03** | **3.35** | **3.78** |
| **Tempe Difference in ^0^K** | **4.67** | **4** | **2.26** | **3.54** | **3.55** | **3.03** | **3.35** | **4** |
| **Isentropic Efficiency** | **2.8** | **2.5** | **1.4** | **2.6** | **1.6** | **0.7** | **1.9** | **1.92** |
| **Adiabatic Efficiency** | **1.89** | **1.68** | **0.94** | **1.61** | **1.2** | **0.61** | **1.32** | **1.4** |
| **COP** | **0.01** | **0.077** | **0.04** | **0.06** | **0.078** | **0.077** | **0.065** | **0.079** |

**Table-S2: 4 bar pressure at inlet of the Vortex tube**

| **L/D Ratio = 40** | |  |  |  |  |  |  |  |
| --- | --- | --- | --- | --- | --- | --- | --- | --- |
|  | **Stainless Steel** | **Brass** | **PVC** | **CPVC** | **Acraulic** | **Nylon** | **Bronze** | **Copper** |
| **Inlet Pressure in Bar** | **4** | | | | | | | |
| **Inlet Temperature in ^0^K** | **303** | **303** | **303** | **303** | **303** | **303** | **303** | **303** |
| **Cold Outlet** | **300.77** | **300.88** | **301.39** | **300.86** | **301.39** | **301.8** | **301.08** | **301.12** |
| **Hot Outlet 1** | **307.03** | **306.67** | **305.56** | **306.06** | **306.61** | **306.66** | **306.31** | **306.79** |
| **Hot Outlet 2** | **307.01** | **306.66** | **305.56** | **306.07** | **306.62** | **306.67** | **306.26** | **306.8** |
| **Inlet - Cold Out** | **2.23** | **2.12** | **1.61** | **2.14** | **1.61** | **1.2** | **1.92** | **1.88** |
| **Hot Out - Cold Out** | **6.25** | **5.79** | **4.18** | **5.21** | **5.23** | **4.86** | **5.23** | **5.67** |
| **Tempe Difference in ^0^K** | **6.25** | **5.79** | **4.18** | **5.21** | **5.23** | **4.86** | **5.23** | **6** |
| **Isentropic Efficiency** | **2.2** | **2.1** | **1.6** | **2.1** | **1.6** | **1.2** | **1.9** | **1.9** |
| **Adiabatic Efficiency** | **1.44** | **1.35** | **0.97** | **1.27** | **1.12** | **0.91** | **1.22** | **1.25** |
| **COP** | **0.116** | **0.109** | **0.09** | **0.06** | **0.104** | **0.106** | **0.09** | **0.115** |

**Table-S3: 6 bar pressure at inlet of the Vortex tube**

| **L/D Ratio = 40** | |  |  |  |  |  |  |  |  |
| --- | --- | --- | --- | --- | --- | --- | --- | --- | --- |
|  | **Stainless Steel** | **Brass** | **PVC** | **CPVC** | **Acraulic** | **Nylon** | **Bronze** | **Copper** |  |
| **Inlet Pressure in Bar** | **6** | | | | | | | | |
| **Inlet Temperature in ^0^K** | **303** | **303** | **303** | **303** | **303** | **303** | **303** | **303** |  |
| **Cold Outlet** | **300.11** | **300.19** | **300.6** | **300.19** | **300.67** | **301.05** | **300.27** | **300.28** |  |
| **Hot Outlet 1** | **307.89** | **307.62** | **306.64** | **307.02** | **307.51** | **307.61** | **307.33** | **307.84** |  |
| **Hot Outlet 2** | **307.87** | **307.61** | **306.64** | **307.03** | **307.52** | **307.62** | **307.28** | **307.85** |  |
| **Inlet - Cold Out** | **2.89** | **2.81** | **2.4** | **2.81** | **2.33** | **1.95** | **2.73** | **2.72** |  |
| **Hot Out - Cold Out** | **7.78** | **7.43** | **6.03** | **6.82** | **6.84** | **6.56** | **7.06** | **7.56** |  |
| **Tempe Difference in ^0^K** | **7.78** | **7.43** | **6.03** | **6.82** | **6.84** | **6.56** | **7.06** | **7.5** |  |
| **Isentropic Efficiency** | **2.3** | **2.3** | **1.98** | **2.3** | **1.9** | **1.61** | **2.2** | **2.2** |  |
| **Adiabatic Efficiency** | **1.49** | **1.44** | **1.19** | **1.34** | **1.26** | **1.12** | **1.37** | **1.4** |  |
| **COP** | **0.141** | **0.133** | **0.116** | **0.116** | **0.13** | **0.1** | **0.125** | **0.141** |  |

**Table-S4: 8 bar pressure at inlet of the Vortex tube**

| **L/D Ratio = 40** | |  |  |  |  |  |  |  |
| --- | --- | --- | --- | --- | --- | --- | --- | --- |
|  | **Stainless Steel** | **Brass** | **PVC** | **CPVC** | **Acraulic** | **Nylon** | **Bronze** | **Copper** |
| **Inlet Pressure in Bar** | **8** | | | | | | | |
| **Inlet Temperature in ^0^K** | **303** | **303** | **303** | **303** | **303** | **303** | **303** | **303** |
| **Cold Outlet** | **299.51** | **299.47** | **299.85** | **299.6** | **299.98** | **300.36** | **299.49** | **299.47** |
| **Hot Outlet 1** | **308.75** | **308.58** | **307.68** | **307.91** | **308.37** | **308.51** | **308.38** | **308.85** |
| **Hot Outlet 2** | **308.73** | **308.57** | **307.68** | **307.92** | **308.38** | **308.52** | **308.33** | **308.86** |
| **Inlet - Cold Out** | **3.49** | **3.53** | **3.15** | **3.4** | **3.02** | **2.64** | **3.51** | **3.53** |
| **Hot Out - Cold Out** | **9.24** | **9.11** | **7.83** | **8.31** | **8.39** | **8.15** | **8.88** | **9.38** |
| **Tempe Difference in ^0^K** | **9.24** | **9.11** | **7.83** | **8.31** | **8.39** | **8.15** | **8.88** | **9.38** |
| **Isentropic Efficiency** | **2.5** | **2.6** | **2.3** | **2.5** | **2.2** | **1.9** | **2.5** | **2.5** |
| **Adiabatic Efficiency** | **1.6** | **1.58** | **1.37** | **1.48** | **1.4** | **1.3** | **1.55** | **1.6** |
| **COP** | **0.166** | **0.161** | **0.135** | **0.142** | **0.17** | **0.155** | **0.166** | **0.168** |

**Table-S5: 10 bar pressure at inlet of the Vortex tube**

| **L/D Ratio = 40** | |  |  |  |  |  |  |  |
| --- | --- | --- | --- | --- | --- | --- | --- | --- |
|  | **Stainless Steel** | **Brass** | **PVC** | **CPVC** | **Acraulic** | **Nylon** | **Bronze** | **Copper** |
| **Inlet Pressure in Bar** | **10** | | | | | | | |
| **Inlet Temperature in ^0^K** | **303** | **303** | **303** | **303** | **303** | **303** | **303** | **303** |
| **Cold Outlet** | **298.95** | **298.82** | **299.17** | **299.06** | **299.35** | **299.7** | **298.75** | **298.7** |
| **Hot Outlet 1** | **309.59** | **309.51** | **308.7** | **308.77** | **309.2** | **309.37** | **309.4** | **309.85** |
| **Hot Outlet 2** | **309.57** | **309.5** | **308.7** | **308.78** | **309.21** | **309.38** | **309.35** | **309.86** |
| **Inlet - Cold Out** | **4.05** | **4.18** | **3.83** | **3.94** | **3.65** | **3.3** | **4.25** | **4.3** |
| **Hot Out - Cold Out** | **10.64** | **10.69** | **9.53** | **9.72** | **9.86** | **9.67** | **10.65** | **11.15** |
| **Tempe Difference in ^0^K** | **10.64** | **10.69** | **9.53** | **9.72** | **9.86** | **9.67** | **10.65** | **11.15** |
| **Isentropic Efficiency** | **2.7** | **2.8** | **2.6** | **2.7** | **2.5** | **2.2** | **2.9** | **3** |
| **Adiabatic Efficiency** | **1.72** | **1.72** | **1.54** | **1.59** | **1.55** | **1.5** | **1.75** | **1.81** |
| **COP** | **0.19** | **0.188** | **0.165** | **0.167** | **0.179** | **0.184** | **0.185** | **0.198** |

**Table-S6: 12 bar pressure at inlet of the Vortex tube**

| **L/D Ratio = 40** | |  |  |  |  |  |  |  |
| --- | --- | --- | --- | --- | --- | --- | --- | --- |
|  | **Stainless Steel** | **Brass** | **PVC** | **CPVC** | **Acraulic** | **Nylon** | **Bronze** | **Copper** |
| **Inlet Pressure in Bar** | **12** | | | | | | | |
| **Inlet Temperature in ^0^K** | **303** | **303** | **303** | **303** | **303** | **303** | **303** | **303** |
| **Cold Outlet** | **298.35** | **298.13** | **298.51** | **298.52** | **298.78** | **299.1** | **298.09** | **298.01** |
| **Hot Outlet 1** | **310.46** | **310.47** | **309.63** | **309.64** | **310.01** | **310.21** | **310.36** | **310.81** |
| **Hot Outlet 2** | **310.44** | **310.46** | **309.63** | **309.65** | **310.02** | **310.22** | **310.34** | **310.82** |
| **Inlet - Cold Out** | **4.65** | **4.87** | **4.49** | **4.48** | **4.22** | **3.9** | **4.91** | **4.99** |
| **Hot Out - Cold Out** | **12.11** | **12.34** | **11.12** | **11.12** | **11.23** | **11.11** | **12.27** | **12.8** |
| **Tempe Difference in ^0^K** | **12.11** | **12.34** | **11.12** | **11.12** | **11.23** | **11.11** | **12.27** | **13** |
| **Isentropic Efficiency** | **3** | **3.16** | **2.9** | **2.91** | **2.7** | **2** | **3.1** | **3.2** |
| **Adiabatic Efficiency** | **1.84** | **1.9** | **1.72** | **1.71** | **1.67** | **1.66** | **1.91** | **1.95** |
| **COP** | **0.21** | **0.216** | **0.19** | **0.192** | **0.2** | **0.2** | **0.21** | **0.22** |

**Table- S7: Main Properties of Materials**

| **Material** | **Copper** |  | **Material** | **Bronze** |
| --- | --- | --- | --- | --- |
| Density | 8940 kg/m^3^ |  | Density | 8700 kg/m^3^ |
| Melting Point | 1084.62^0^C |  | Melting Point | 950^0^C |
| Modulus of Elasticity | 117 GPa |  | Modulus of Elasticity | 120 GPa |
| Thermal Conductivity | 386 W/m.K |  | Thermal Conductivity | 26 W/m. K |
|  |  |  |  |  |
| **Material** | **Brass** |  | **Material** | **SS** |
| Density | 8440kg/m^3^ |  | Density | 7500 kg/m^3^ |
| Melting Point | 916^0^C |  | Melting Point | 1450^0^C |
| Modulus of Elasticity | 103.4 GPa |  | Modulus of Elasticity | 193GPa |
| Thermal Conductivity | 116 W/m. K |  | Thermal Conductivity | 15 W/m. K |
|  |  |  |  |  |
| **Material** | **CPVC** |  | **Material** | **PVC** |
| Density | 1450 kg/m^3^ |  | Density | 1467kg/m^3^ |
| Melting Point | 150^0^C |  | Melting Point | 260^0^C |
| Modulus of Elasticity | 2.52 - 3.10 GPa |  | Modulus of Elasticity | 0.00300 - 4.83 GPa |
| Thermal Conductivity | 0.139W/m. K |  | Thermal Conductivity | 0.33W/m. K |
|  |  |  |  |  |
| **Material** | **Acraulic** |  | **Material** | **Nylon** |
| Density | 1.19 kg/m^3^ |  | Density | **1.15 kg**/m^3^ |
| Melting Point | 160^0^C |  | Melting Point | 220^0^C |
| Modulus of Elasticity | 3.2 GPa |  | Modulus of Elasticity | 2.7 GPa |
| Thermal Conductivity | 0.2W/m. K |  | Thermal Conductivity | - 1. W/m. K |
